# Effectiveness of Conservative Treatment According to Severity and Systemic Disease in Carpal Tunnel Syndrome: A Systematic Review

**DOI:** 10.3390/ijerph18052365

**Published:** 2021-02-28

**Authors:** Mar Hernández-Secorún, Raquel Montaña-Cortés, César Hidalgo-García, Jacobo Rodríguez-Sanz, Jaime Corral-de-Toro, Sofia Monti-Ballano, Sami Hamam-Alcober, José Miguel Tricás-Moreno, María Orosia Lucha-López

**Affiliations:** 1 Physiotherapy Department, Faculty of Health Sciences, Universidad de Zaragoza, 50009 Zaragoza, Spain; marhsecorun@unizar.es (M.H.-S.); 722162@unizar.es (R.M.-C.); 682825@unizar.es (J.C.-d.-T.); 681265@unizar.es (S.M.-B.); jmtricas@unizar.es (J.M.T.-M.); orolucha@unizar.es (M.O.L.-L.); 2 Unidad de Investigación en Fisioterapia, University of Zaragoza, 50009 Zaragoza, Spain; 3 Department of Basic Sciences, Faculty of Medicine and Health Sciences, Universitat Internacional de Catalunya, 08195 Sant Cugat del Vallès, Spain; jrodriguezs@uic.es; 4 ACTIUM Functional Anatomy Group, Universitat Internacional de Catalunya, 08195 Sant Cugat del Vallès, Spain; 5 Unit of Reconstructive Surgery of the Locomotor System, Hand-Microsurgery, Department of Orthopaedic Surgery and Traumatology, Hospital Universitario Miguel Servet, 50009 Zaragoza, Spain; jshamam@salud.aragon.es

**Keywords:** carpal tunnel syndrome, conservative treatment, diabetic neuropathy, electric stimulation therapy, musculoskeletal manipulation, pain

## Abstract

(1) Background: Carpal tunnel syndrome (CTS) is the most common peripheral neuropathy in the upper extremity. Conservative treatment has been effective for mild and moderate idiopathic CTS. However, severe CTS and systemic conditions were an exclusion criterion from the studies. The aim of this study is to review the effectiveness of conservative treatment in patients with CTS regardless of the level of severity and the presence or not of systemic diseases in the last ten years. (2) Methods: Randomized controlled clinical trials that compared the effect of conservative treatment on the Boston questionnaire and pain were selected. PubMed, PEDro, Scopus, Cochrane, and Web of Science databases were used. PRISMA statement checklist was performed. (3) Results: 876 studies were recorded, 29 were selected. Pharmacology, Electrotherapy and Manual Therapy had benefits for CTS. Electrotherapy and manual therapy could be effective for severe CTS patients with a systemic condition in the short term, but there was a low percentage of these patients included in the studies. (4) Conclusion: Some pharmacological treatments, manual therapy and electrotherapy have shown benefits for handling CTS, although the most effective combination of techniques is unknown. It would be necessary to include patients with systemic conditions in the selection criteria for future studies.

## 1. Introduction

Carpal tunnel syndrome (CTS) is defined as an entrapment of the median nerve in the carpal tunnel in the wrist, generally associated with an increase in carpal tunnel pressure [1]. It is the most common peripheral neuropathy in the upper quadrant. The prevalence for CTS ranges from 3.8% to 4.9%, with women being three times more predisposed than men [1,2]. The elevated prevalence of this pathology causes annual healthcare costs to rise, increasing the socioeconomic costs [3].

CTS is characterized by pain and paresthesia in the distributions of the median nerve, including the palmar side of the first finger, the second and third finger and the radial half of the fourth finger; it also involves a loss of sensitivity, manual dexterity and functionality. However, the signs and symptoms vary and do not correlate with the level of severity [1,4]. In severe cases, weakness of median nerve innervated muscles and spreading symptoms to the forearm, upper arm and sometimes to shoulder could be described. Atrophy of the thenar eminence and weakness of thumb abduction and opposition seem to be the most significant signs [1,5].

Some clinical conditions such as obesity, diabetes and hypothyroidism are linked to a double risk of developing CTS, due to the increased intraneural pressure in the carpal tunnel or vascular deficiencies [6,7,8]. Different mechanisms have been hypothesized: impaired microvascular circulation for diabetes type 1 and 2 [9]; mucopolysaccharide complex depot for thyroid diseases [10]; hormonal alteration and oedema for menopause and pregnancy [5]; alteration of the fluid balance in the body for obesity [11]. However, more investigation should be done to prove those mechanisms. 

In clinical practice guidelines, conservative treatment is recommended for mild and moderate cases, while a surgical approach is recommended for patients with severe CTS [4]. When dealing with patients having severe CTS without diabetic neuropathy, based on thenar muscle atrophy and electrophysiological findings, surgical release is recommended [4]. Even if the results of surgical intervention (carpal tunnel release) are predictable to some extent: about 80–90% of patients have good to excellent long-term outcomes, remaining 10–20% have less satisfactory (suboptimal) outcomes, and some of them poor outcome [12]. However, severe symptoms could be present without electrophysiological abnormalities. In a systematic review [13], the effectiveness of the conservative treatment in patients with mild and/or moderate CTS was showed. The use of manual therapy, electrotherapy and pharmacological treatments (the most common techniques nowadays) was described, but no conclusion was given as to which of them was more effective. Other systematic review shows that conservative treatment is effective for short terms but not to longer terms and finally resorting to surgical intervention [14]. These types of treatment were reviewed with the exclusion of patients having severe intensity and/or systemic pathology, conditions that are usually a criterion for exclusion in the sample selection. However, the CTS prevalence in patients with diabetes ranges from 14% in subjects without diabetic neuropathy, up to 30% in patients with diabetic neuropathy [15], with a 84% of risk from suffering CTS during their lifetimes [7]. In addition, even if patients with diabetic neuropathy may improve function and symptoms after CTS surgical release, these subgroup of patients have worst results than CTS patients without this condition [16]. Moreover, they suffer from more complications arising from those procedures. These aspects should be taken into account for treatment election [5,9] for this type of patients and conservative approach could be considered.

We decided to carry out this systematic review because less attention is given to the conservative approach in patients with severe CTS and systemic diseases, there is elevated incidence and prevalence of systemic pathology in CTS, and the fact that CTS involves high individual and socioeconomic costs. 

The aim of this study was to review the effectiveness of the conservative treatment in patients having CTS, including severe CTS and patients with systematic diseases in the last 10 years. 

## 2. Materials and Methods

### 2.1. Protocol and Register

A systematic review based on the Preferred Reporting Items for Systematic Reviews and Meta-Analyses (PRISMA) statement checklist [17] was designed. The systematic review was registered on the Open Science Framework digital platform: https://osf.io/45q28/ (accessed date 17 February 2021) (DOI 10.17605/OSF.IO/45Q28).

### 2.2. Information Sources and Search

To create an accurate search strategy, the PICOS (Population, Intervention, Comparison, Outcome and Study design) strategy was taken into account. The Population was people suffering from CTS; the Intervention was conservative treatment such as manual therapy, exercise, electrotherapy or pharmacology; the Comparison was Control, Placebo or other therapies; the Outcome was pain and function; and the Study Design was Randomized Controlled Trial. The following databases were searched: PubMed, Physiotherapy Evidence Database (PEDro), Cochrane Library, SCOPUS and Web of Science. The MESH terms used were “Carpal Tunnel Syndrome”, “Therapy”; “Manual Therapy”; “Neural Mobilization” and “Treatment Outcome”. Common filters used were language, document type and years. Different search strategies were performed for each database, according to their own filters. The search in PEDro was more concise than the others due to its search method. Boolean operators were used to limit or enlarge the strategy research. Boolean “OR” was used to enlarge the search strategy linking similar terms. Boolean “AND” was used to connect different terms that should be included in search studies in order to limit the search strategy. Table 1 shows the search strategy from each database. The search was performed for dates between 6 September–2 October 2020. 

### 2.3. Eligibility Criteria

The selection of articles included all studies published since 2010 that fulfilled the following criteria: 1. Randomized controlled clinical trials that compared a conservative treatment group with a control group, a placebo group or a group that received a strictly different treatment; 2. studies in which the sample included patients diagnosed with idiopathic-type CTS with or without systemic pathology; 3. studies including pain and functional variables; 4. studies that evaluated a functional item using the Boston Carpal Tunnel Questionnaire (BCQT), a tool with standardized, patient-based results for the seriousness of the symptoms and the functional status in patients with CTS [18]; 5. studies having a score equal to or more than 6 in the PEDro Scale; 6. studies published in English, French or Spanish.

We excluded the studies in which the sample included the following criteria: 1. pregnant women; 2. patients having suffered traumatisms in the cervical area or upper extremity that might contribute to CTS and its symptoms; 3. subject with previous surgery and/or surgery during the study period as a CTS treatment. 

### 2.4. Study Selection

Eligible articles and data extraction were conducted independently by two authors (M.H.S. and R.M.C.). Firstly, they evaluated titles and abstracts following the criteria for eligibility, and then complete texts. For any discrepancies or doubts, a third author (C.H.G.) was consulted to resolve the discrepancy.

### 2.5. Data Extraction Process

The following information was extracted for each study: (1) variables analyzed; (5) follow-ups; and (6) results. 

### 2.6. Risk of Bias in the Individual Studies

To assess the methodological quality of the clinical trials, the PEDro scale and the Risk of Bias 2 (RoB2) tool in the Cochrane database were used. 

The PEDro scale is based on the Delphi list, based on an expert consensus, to help the reader to identify the clinical trials with sufficient internal validity and sufficient statistical information to make their results interpretable. It is formed by 11 criteria to answer using a “Yes” or “No” response, as long as the information is clearly expressed in the study. Each criterion is given 1 point, and the maximum score is 11 points [19].

The RoB2 tool is the second version of the Cochrane tool to assess the risk of bias in clinical trials. The biases are evaluated in 5 domains: (1) randomization process; (2) effect of being assigned to intervention; (3) missing outcome data; (4) measurement of the outcome; (5) reported results. Within each domain, 1 or more questions must be answered. These answers lead to the judgements of “low risk of bias” “some concerns” or “high risk of bias” [20].

## 3. Results

### 3.1. Study Selection

The literature search yielded 876 articles (PubMed: 155, Web of Science: 109; Scopus: 313; PEDro: 62; Cochrane: 237). After we eliminated the duplicated studies, 226 articles were selected for further analysis.

The first analysis focused on the study title and the abstract. We excluded 115 articles because of the study design, sample, presence of surgical intervention, lack of complete article or language different from those of the inclusion criteria.

**Figure 1:**
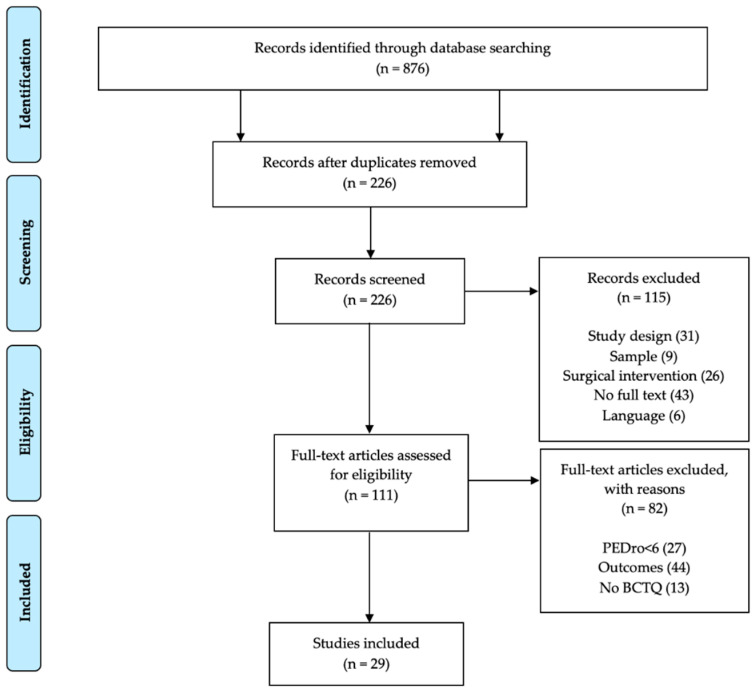
Flow Diagram.

The last step was analyzing the remaining 111 complete articles to select the ones that fulfilled the inclusion criteria, being finally selected 29 articles for analysis. The selection process is shown in the flow diagram (Figure 1). 

### 3.2. Study Characteristics

#### 3.2.1. Sample

The study characteristics are presented in Table 2, Table 3 and Table 4. All the articles offered a sample of 1780 upper limbs with CTS. Of these, 28 articles assessed patients with idiopathic CTS, 2 also assessed patients with hypertension and diabetes [21,22] and only 1 article assessed strictly diabetic patients [23]. Additionally, all the studies include mild and moderate severity of CTS, and 8 studies included severe CTS patients [22,23,24,25,26,27,28,29]. 

To diagnose the presence of CTS and classify the intensity level of the CTS syndrome, most of studies performed an electrophysiological study. However, three of them did not include any neurological study, using only physical examination [25,30,31]. Mostly, the physical examination consisted of the presence of symptoms such as paresthesia and pain in median nerve distribution area, positive Phalen’s and Tinel’s test. Others test performed was Katz’s diagram and the flick sign. The Guideline of the American Academy of Neurology (AAN) was used to classify CTS severity according to neurophysiological findings.

#### 3.2.2. Intervention 

Six studies observed the effectiveness of drugs [29,32,33,34,35,36]; eight studies evaluated the effectiveness of electrotherapy [21,22,25,37,38,39,40,41] (diathermy, shock waves, radiofrequency, and so on); and eight studies the effect of manual therapy [26,27,28,42,43,44,45,46]. Three studies compared electrotherapy and a drug intervention [24,47,48] and two studies compared electrotherapy and manual therapy [23,30]. Two articles differed from these categories: one study analyzed the effectiveness of an alternative therapy (lavender ointment) [49] and the other one, a thermal agent (paraffin wax) [50]. Twelve studies had a control group [22,25,27,28,33,41,42,43,44,45,46]; nine studies a placebo group [21,29,37,38,39,40,42,48,49]; and thirteen studies an alternative treatment [23,24,26,27,30,32,34,35,36,37,39,47,48]. There were six articles having three or more groups [27,32,37,39,42,48].

Applying a splint, applied during night and/or day, was generally the control treatment of choice. The material of the splint was neoprene or a thermoplastic tissue. Some studies provided costumed or prefabricated splints, without describing the kind of material, except for studies that used kinesiotaping. The wrist was placed in neutral position, but this position was only described in two studies (0–5 degrees of wrist extension) [33,44]. The educational instruction to explain how to use the splint was considered in a few studies. Only one study combined basic medical care such as ibuprofen with splint treatment [44].

Regarding pharmacological treatment, the studies compared a type of corticosteroids with other drugs or with placebos. There were no statistically significant conclusions as to their effectiveness. The studies that analyzed using platelet-rich plasma did not reveal significant differences [33,34]. Combining corticosteroids and night splints presented statistically significant improvements compared with using solely drugs or night splints. Electrotherapy presented statistically significant improvements over corticosteroids [47]. The most often used drugs were triamcinolone, lidocaine and methylprednisolone. One dose of 5% dextrose was statistically significant compared to placebo or triamcinolone. 

Concerning electrotherapy, all the outcomes showed statistically significant improvements in pain and/or function (BCQT) against placebo or control groups. Most of these studies added a night splint as part of the treatment. In isolation, electrotherapy was not as effective compared with the control and/or placebo groups. The therapies used were shock waves [21,24,47], ultrasound [37,48], laser [38,39], electro-acupuncture [25,41], diathermy [40] and radiofrequency [22]. 

As far as manual therapy is concerned, the articles presented significantly better improvements in these groups in functional variables (BCQT), and some, in symptoms and strength. These studies were the main ones obtaining changes in the electrophysiological parameters. The studies that compared manual therapy and electrotherapy found significant differences in favor of the manual therapy group [23,30]. The interventions featured the use of neurodynamic techniques [26,27,45], acupuncture [28,44], exercise [50], joint mobilization [43,46] or a combination of various techniques [23,30,42]. Nerve mobilization or neurodynamic techniques were described differently among the studies. Three studies included mobilization from shoulder to fingers, described by Shacklock [23,30,45] as initial position; shoulder abduction to 90°; arm external rotation; wrist and fingers extension; forearm supination; and elbow extension, compared to those who performed only wrist and hand mobilization [27,40] in six different positions as grasping, finger extension, wrist extension, thumb extension, forearm supination, and gentle stretch of the thumb with the opposite hand. Joint mobilization consisted in performing distraction and dorsal/palmar glides on the first row of the radiocarpal joints [43] or dorsal and palmar glide on scaphoid and hamate bones [46]. Both studies aimed to improve wrist range of motion on CTS patients. In addition, treatment of soft tissue was complementary to other manual techniques. Acupuncture techniques were described in isolation. Both studies followed a similar protocol, involving described acupoints unilaterally for the involved side. Hadianfard et al. [44] include nine acupoints compared to Tezel et al. [28] that performed the therapy on six acupoints. Functional massage (a combination of muscle compression while mobilizing the joint) of the soft tissue was performed in two studies, complementary to other manual therapy treatment. Talebi et al. [23] performed it on pronator teres muscle, unlike Wolny et al. [30] who performed it on the trapezius muscle. Both techniques were not described. 

Lastly, lavender ointment combined with a night splint did not yield any significant differences compared with the placebo groups [49]. Lavender ointment and placebo were instructed to be applied on their affected wrist in the morning and evening time for 40 days. 

#### 3.2.3. Variables

All the studies included assessed function using the BCTQ scale, and 3 added the DASH questionnaire on shoulder, elbow and hand impairment [25,26,27]. All the articles evaluated pain, with 25 using a visual analogic scale (VAS) and 4 using the numeric pain rating scale (NPRS) [25,26,43,45]. In addition to the evaluation of pain, 3 articles used a VAS to quantify paresthesia [21,34,37].

Twenty articles analyzed electrophysiological parameters, 12 articles analyzed grip strength and/or pinch strength [21,22,25,26,32,37,39,42,43,45] and 6 articles analyzed the cross sectional area of the carpal tunnel using a ultrasound-guided procedure [21,22,29,34,36,37]. Other variables measured were quality of life (Nottingham Health Profile [NHP] and World Health Organization Quality of Life [WHOQOL]), neurodynamic tests, range of movement, or clinical parameters (Tinel and Phalen tests).

#### 3.2.4. Follow-Up

Most studies had a follow-up of no more than 6 months, with the majority of the studies having short- and mid-term follow-ups. There were 24 articles that studied the short-term (≤3 months) effects, while 5 had mid-term (≤6 months) follow-ups. The studies analyzing drug application mainly analyzed mild term follow-ups.

#### 3.2.5. Evaluation of the Risk of Bias

The methodological quality scores ranged from 6 to 10. The mean quality of all the studies analyzed by the PEDro scale [19] was 7.5 over 11. 14 of the 29 studies obtained a score of 8 or more on this scale (Table 5). The blinding of the participants of the samples, the blinding of the researcher who carried out the therapy were the criteria with no achievement throughout the studies. Moreover, the criterion of “all subject for whom outcome were available received the treatment or control condition as allocated or, where this was not the case, data for at least one key outcome was analyzed by intention to treat” was less achieve by the studies.

**Figure 2:**
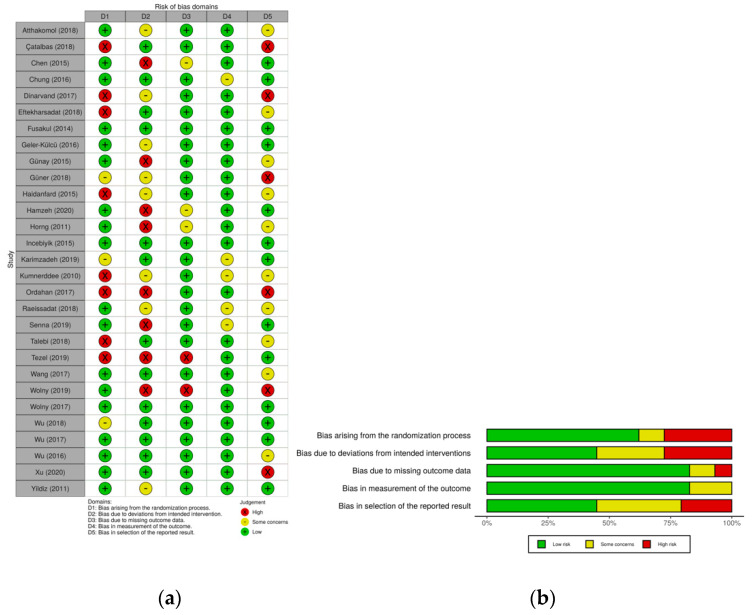
(**a**) Summary of Risk of Bias 2.0. (**b**) Risk of bias 2.0. graph.

The RoB2 tools [20] showed that the aspects with the worst methodological quality in all the studies are found in the effect of assigning to the intervention and the results reported. Variable measurement seems to have the best methodical quality in all the studies considered (Figure 2a,b). Even though, it seems that good methodological quality was performed in the majority of studies.

## 4. Discussion

Our study objective was to review the effectiveness of conservative treatment in patients with CTS of any level of severity, with the inclusion of patients with systemic conditions. In patients having mild or moderate intensity, three types of treatment were used: drug, electrotherapy and manual therapy. Severe patients were not analyzed isolated, not allowing which kind of treatment is effective for them. Patients with systemic disease were poorly represent in samples. Two studies include patients with diabetes and hypertension, and another analyzed aa sample of diabetic patients. Manual therapy could be effective for those patients at short-term, but more future studies have to be done.

Splint therapy was the more used control or complementary intervention. Adding a wrist splint to all kind of intervention treatments seem to be more beneficial than not including the orthosis. Hall et al. [51] observed that conservative treatment program including full-time splinting and formal education can improve symptoms and function on CTS patients. It could be considered for symptom reduction in all patients waiting for the surgical intervention. Burke et al. [52] observed the angle of the splint does make a difference in subjective relief of the symptoms of carpal tunnel syndrome, and that this difference is apparent after wearing the splints for only two weeks. They prove that the neutral angle wrist splint will provide better symptom relief than the traditional 20° extension wrist splint or even in flexion. Even though, a customized orthosis should be the best option for the most effective treatment of the patient’s symptoms.

Within the pharmacological treatment, using local corticosteroids, as methylprednisolone, was common. Oral corticosteroids reduce oedema, improving the space between the carpal tunnel and the median nerve [5]. It shows to have strong evidence to improve reported patients outcome [53]. Solely a greater dose of corticosteroids was found to be statistically significant for function after three months [32]. However, it was not more effective than shock waves after 24 weeks [24], or than a 5% dextrose solution after 6 months, at the level of symptoms and function [36]. Its effect was more beneficial combined with a splint therapy than solely with a splint after 12 weeks, at the level of function, symptoms and electrophysiological parameters [54], with a fairly good quality of studies. Injection of dextrose is a new treatment for peripheral neuropathies, because it possesses osmolarity similar to that of normal saline, and no harmful effects have been reported from animal and human studies [55]. Furthermore, one dose of 5% dextrose showed to be effective to improve function and CTS symptoms. Similar to a glucose molecule, the mechanism of an injection of hypo-osmolar 5% dextrose postulated induced analgesic effect on tender peripheral nerves as well as central nerve system by osmotic rupture of local cells [56,57]. Another treatment growing in popularity is injecting platelet-rich plasma. This is used as an alternative to surgery because of the regenerative effect it can produce in patients with demyelination and axonal degeneration in CTS [48]. Platelet-rich plasma seems to be more effective three months after the injection at the level of symptoms, function and electrophysiological and clinical parameters than methylprednisolone injection [34]. However, there are no evidences indicating that it is more effective than night splinting after 10 weeks of treatment [33].

Considering electrotherapy, few studies assessed its effectiveness during its isolated application rather than combined with other type of therapies (manual therapy, splint, bandaging). This makes it difficult to evaluate the effectiveness of electrotherapy alone. Electro-acupuncture was the only treatment compared alone with night splinting; it obtained superior improvements in pain following five weeks of treatment compared with splinting [41]. However, the quality of the study was moderate. Low-power laser combined with bandaging [39], the use of diathermy and nerve gliding exercises [40] and ultrasound with ketoprofen gel and night splinting [48] presented better improvements in symptoms and function following treatment than these procedures alone or compared with placebo.

Focusing on manual therapy, all the techniques were assessed in combination or with other types of treatment (splints, bandages, diathermy, exercises). It seems that carpal bone mobilizations combined with night splinting are more effective after treatment at the level of function and symptoms than splinting alone. However, the methodological quality of these studies was moderate. It also seems that neurodynamic techniques used alone improve electrophysiological variables of function and symptoms compared with a control group, with moderate methodological quality [28]. Furthermore, neurodynamic techniques combined with other therapy, soft tissue or joint mobilization was more effective in symptoms and function following treatment than the combination of transcutaneous electrical nerve stimulation (TENS) and ultrasound or laser and ultrasound [30]; following treatment, acupuncture combined with splinting seems to be more effective for symptoms than splinting alone or combined with ibuprofen [28,44]. Acupuncture plus splinting also improved electrophysiological variables and function, in a study having good methodological quality [44]. Following treatment, paraffin combined with splinting and exercises seems to have a positive effect on function and electrophysiological parameters, but the study had moderate methodological quality [50]. All these treatments seem to have short term treatment effects, but few studies assessed their mid or long-term effect. Consequently, their effect on resolving CTS cannot be established. 

The variability while performing the different manual techniques between studies can explain the diversity of results. Firstly, there is a lack of terminology consensus or standardization in the techniques whose aim is to mobilize the nervous system. Moreover, parameters such as the mobilization dosages, the number of joints to be mobilized and the consideration to stabilize or not the wrist joint while performing the gliding mobilization techniques are not uniform throughout the studies. Shacklock (2005) considers the stabilization of the related joint to the nerve region to be mobilized important to achieve a specific mobilization. On the other hand, joint mobilization aims to improve wrist range of motion. However, some joint mobilizations [58], described as transverse and ventral glide on dorsal side of the first carpal row, have been designed to release carpal tunnel syndrome, increasing cross-sectional area of carpal tunnel. In addition, soft tissue mobilization aims to reduce pressure on the carpal tunnel syndrome by improving the mobility of the myofascial tissues adjacent to the nerve. More studies need to be carried out to see the most effective combination of soft tissue and joint mobilization treatment in CTS patients. 

Lavender oil is frequently used as an herbal medication for a wide variety of diseases, either topically or by inhalation. In vivo studies have shown that the active ingredients of lavender oil have important anti-inflammatory, anti-nociceptive, sedative, and analgesic therapeutic potentials [59]. However, using lavender ointments combined with night splints was not any more effective than a placebo ointment combined with splinting [49]. Results were only reported pre- and post-intervention, without follow-ups. 

When severe patients were included, the isolated effect that conservative treatments produce in these patients was not found. These patients with electrophysiological abnormalities could show thenar muscle atrophy, and more diffuse symptoms. Surgical approach was the option of choice [5]. In some studies, electrotherapy was applied in mild, moderate and severe cases. Combined with a night splint, it seemed effective for symptoms and function following treatment; however, it is unclear whether these effects will be maintained at long-term [22,25]. In patients with severe CTS, other studies reported the effect of manual therapy including nerve gliding exercises [26], tendon gliding exercises [27] and acupuncture [28]. Both tendon and nerve gliding exercises improved function immediately and six months after treatment but combined with paraffin [27] and splinting or an exercise program [26]. Acupuncture reduced pain following treatment, once again combined with night splinting [28]. The moderate quality of these studies and the combination with other types of treatment make it difficult to establish the best effect of these therapies on the more severe CTS cases. It would be interesting to report results stratifying level of CTS severity to prove that conservative management it is a beneficial option for these patients. Still, electrotherapy and manual therapy could be beneficial to severe CTS.

Diagnosis of CTS should be performed by physical examination and nerve conduction studies [53]. All the studies included in this review performed one or both items. Electrophysiological studies were considered the gold standard to diagnose CTS patients. These tests can define the degree of demyelination and axonal loss that has occurred. Bland [60] scale described degrees to classify the severity of CTS as: Grade 0 denotes no neurophysiological abnormality; Grade 1, very mild CTS, detected only in two sensitive test; Grade 2, mild CTS as orthodromic sensory conduction velocity from index finger <40m/s with motor terminal latency from wrist to abductor pollicis brevis (APB); Grade 3, moderately severe CTS as motor terminal latency >4.5 ms and <6.5 ms with preserved index finger sensory nerve action potential (SNAP); Grade 4, severe CTS as motor terminal latency >4.5 ms and <6.5 ms with absent SNAP; Grade 5, very severe CTS as motor terminal latency >6.5 ms; and Grade 6, extremely severe CTS as surface motor potential from APB <0.2 mV peak-to-peak. However, the American Association of Orthopedic Surgeon [61] bundle this classification in mild (grade 1 and 2), moderate (grade 3) and severe (grade 4, 5, and 6), as reviewed studies performed. In the other hand, physical examination was performed in all studies reviewed, nonetheless a variety of test was performed. Phalen test, Tinel test and symptoms in neuroanatomical median nerve distribution were the most performed. Similarly, to electrophysiological studies, the physical examination tends to test the myelinated fiber function. Nowadays, this criterion should be disputed due to negative electrophysiological parameters in presence of CTS or symptoms beyond neuroanatomical distribution [7]. A new approach proposed to include the assessment of small nerve fibers or unmyelinated nerve fibers such as quantitative sensory testing to help confirming the presence of CTS [62]. This includes assessment of heat, cold and vibration sensation thresholds to evaluate nerve fibers that are not tested during nerve electrophysiological studies. Besides, combination of all these items must be taken into consideration in order to make a correct diagnosis, relating it to the patient’s clinical history.

Another point is that, although CTS is generally idiopathic, some conditions such as diabetes or hypertension predispose individuals to suffer from neuropathy. In these patients, the risk of suffering CTS is three times greater than in healthy patients [63]. However, in studies including patients with hypertension and/or diabetes (only 3 of 29), they only represented from 5% to 20% of all the patients evaluated [21,22]. Consequently, the direct effect of electrotherapy in patients with hypertensions and/or diabetes cannot be established. There was only a single study in the review [23] that studied patients with diabetes and CTS. It was found that, after four weeks of treatment, manual therapy (soft tissue and nerve mobilization) seemed more effective than the use of TENS and ultrasound on function, symptoms and neurodynamic results. However, this study did not present long-term results and its methodological quality was only moderate. Assessing small fibers nerve could help therapist to perform a more comprehensive approach on patients with both CTS and systemic condition. Even though, a combined protocol of manual techniques could be more effective for diabetic patients with CTS than electrotherapy in the short term.

We believe that there is insufficient research on CTS in patients with systemic pathology. There is a lack of representation of CTS patients with diabetes in the studies given their high prevalence and the possibility of developing CTS throughout their lives. Moreover, the conservative approach should be more studied in this subgroup as it can be more difficult to diagnose CTS in patients with diabetes because the earliest symptoms can be mistaken for diabetic polyneuropathy [64], and controversy exists about surgical treatment for these patients [65]. Given the lack of representation of patients with CTS and systemic diseases in the studies dealing with conservative therapy approaches, we consider this type of patients should be included in the sample selection criteria in studies on conservative treatment for CTS. This would help to ascertain the effect of conservative treatment in more representative CTS samples. 

There are some limitations in this review that arise from the biases found in the articles. The lack of uniformity in the sample selection criteria makes it difficult to compare data among them. The variability of techniques proposed, and their combinations do not permit accurate comparison of their effectiveness either.

The lack of uniformity in methodological quality also hinders accurately evaluating treatment effectiveness. In addition, there might be references in other language besides Spanish, English and French that were not considered due to the language restrictions established in our selection criteria. More reviews should be performed performing quantitative analysis to prove specific results. 

## 5. Conclusions

In patients with mild and moderate CTS, electrotherapy seems to be effective following treatment as long as it is combined with splinting, but its effectiveness is no greater than manual therapy. Corticosteroid injection does not appear to be more effective than saline solution or other techniques such as electrotherapy or manual therapy. No conclusions can be reached on the effectiveness of platelet-rich plasma to treat CTS; further studies are needed. One injection of 5% dextrose seems to be effective for CTS symptoms and function. Wrist splint could be useful to complement treatments, however wrist position should be described in future studies, taking into account the position that best relieves patient’s symptoms. Electrotherapy and manual therapy could be beneficial for severe CTS, and manual therapy for diabetic CTS in the short term. However, the specific effect of conservative treatment should be established with a stratification analysis depending on the intensity level of CTS. More long-term studies need to be carried out to specify which treatment is most beneficial for which type of severity. It seems that some pharmacological treatments, manual therapy and electrotherapy have benefits for handling CTS, although the most effective combination of techniques is unknown. However, it does seem that a combined treatment for each patient might be the most effective option.

A final point is that patients with systemic conditions and severe CTS should be included in the inclusion criteria for future CTS studies analyzing conservative approaches.

## Figures and Tables

**Table 1:** Search Strategy.

Database	Search Strategy	Filters
PubMed	“Carpal Tunnel Syndrome” AND (Therapy OR “Physical Therapy” OR “Manual Therapy” OR “Neural mobilization”) AND Treatment outcome	Text availability: Full text. Article type: Clinical Trial; Randomized Controlled Trial. Publication date: 10 years. Language: English, Spanish, French.
Web of Science	“Carpal Tunnel Syndrome” AND (Therapy OR “Physical Therapy” OR “Manual Therapy” OR “Neural mobilization”) AND Treatment outcome	Document type: Article Timespan: 2010 to 2020
SCOPUS	“Carpal Tunnel Syndrome” AND (Therapy OR “Physical Therapy” OR “Manual Therapy” OR “Neural mobilization”) AND Treatment outcome	Document type: Article Years: 2010–2020 Language: English, Spanish, French
Cochrane Library	“Carpal Tunnel Syndrome” AND (Therapy OR “Physical Therapy” OR “Manual Therapy” OR “Neural mobilization”) AND Treatment outcome	Years: 2010–2020 Trials Language: English, Spanish, French
PEDro	“Carpal Tunnel Syndrome” “Therapy”	Published since: 2010. When searching: Match all search terms (AND). Method: Clinical Trial.

**Table 2:** Characteristics of Studies: Mild and Moderate CTS.

Study	Sample		Groups	Outcomes	Follow-ups	Results
	No. (M/F)	CTS Characteristics				
**Çatalbas** **(2018)**	54 (8/46)	Mild and moderate. Idiopathic	G1: Continuous ultrasound continuo (10 sessions × 10 min) + Night splint (2 wks) G2: Pulsed ultrasound (10 sessions × 10 min) + Night splint (2 wks) G3: Placebo (Ultrasound–10 sessions × 10 min) + Night splint (2 wks)	*BCTQ*, *VAS* (pain and paraesthesia), Grip strength, DSL, DML, SNCV, MNCV, CSA	0, 2, 6 weeks	G1: Improvement in all parameters at 2 and 6 wks [<0167] G2: Improvement in all parameters at 2 and 6 wks [<0167] G3: Improvement in all parameters at 2 and 6 wks [<0167] except for grip strength at 6 wks and DML at 2 wks.
**Dinarvand** **(2017)**	37 (0/37)	Mild and moderate. Idiopathic	G1: Unciform and scaphoid bone mobilization (10 min, 3 v/wk × 8 wks) + Night splint (8 wks) G2: Control (Night splint—8 wks)	*VAS* (pain), *BCTQ*, DSL, DML, SNAP, motor initiation latency.	0, 10 weeks	G1: Improvement in BCTQ and VAS [<05]; DSL y DML [<05]. G2: Improvement in BCTQ and VAS [<05]; DSL y DML [<05]. G1 vs. G2: Improvement in BCQT and VAS G1 [01].
**Eftekharsadat** **(2018)**	48 (7/21)	Mild and moderate. Idiopathic	G1: Lavender ointment (2 times/day × 40 days) + Night splint (40 d) G2: Placebo (ointment 2 times/day × 40 + night splint (40 d)	*BCTQ*, *VAS* (pain), Pinch and grip strength, CMAP, SNAP	0, 40 days	G1: Improvement in BCTQ, VAS, pinch and grip strength [05]. G2: Improvement in BCTQ, VAS, pinch and grip strength [05]. G1 vs. G2: Improvement in means in G1, ls pinch strength [05].
**Fusakul** **(2014)**	112 (4/108)	Mild and moderate. Idiopathic	G1: Diode laser (15 sessions × 5 wks, 3 times/wk) + Splint (12 wks morning and night) G2: Placebo (Laser–15 sessions x 5 wks, 3 times/wk) + Splint (12 wks morning and night)	*VAS* (pain), *BCTQ*, Pinch and grip strength, Treatment evaluation, DSL. SNAP, DML, CMAP	0, 5 and 12 weeks	G1: Improvement in VAS, BCTQ and grip strength at 5 and 12 wks [05]; Pinch strength and DML at 12 wks [05]. G2: Improvement in VAS and BCTQ at 5 and 12 wks [05]. Pinch strength and DML at 12 wks [05] G1 vs. G2: Improvement in BCTQ-SSS at 5 wks [031].
**Geler-Külcü** **(2016)**	60 (2/58)	Mild and moderate. Idiopathic	G1: Kinesio-tape (1 time/5 days with 2d rest) + Gliding exercises (4 wks) G2: Placebo (kinesio-tape–1 time/5 days with 2 days of rest) + Gliding exercises (4 wks) G3: Night splint (as often as possible) + Gliding exercises (4 wks)	VAS (pain), DN4 Questionnaire, BCTQ, Grip strength.	0, 4 weeks	G1: Improvement in VAS, DN4, BCTQ [001]. G2: Improvement in VAS, DN4, BCTQ-SSS [001]. G3: Improvement in VAS, DN4, grip strength, BCTQ-SSS [001]. G1 vs. G3: Significant difference in favour of G1 BCTQ [008].
**Günay** **(2015)**	40 (–/–)	Mild and moderate. Idiopathic	G1: Carpal bone mobilization (10 min/day × 3 times/wk × 10 d) + Splint G2: Control (Splint)	NPRS (Pain), BCTQ, Pinch and grip strength, DSL, DML, SNAP, CMAP, MNCV, MCV, EMG.	0, 3 months	G1: Improvement in all clinical variables [<022]. DSL y SNAP [05]. G2: Improvement in BCTQ-SSS and NPRS [01]. G1 vs. G2: Greatest improvement in G1 grip strength [04] y BCTQ-FSS [01].
**Güner** **(2018)**	63 hands (4/33)	Mild and moderate. Idiopathic	G1: Low-intensity laser (5 times/wk × 3 wks × 12 min) G2: Low intensity laser (5 times/wk × 3 wks × 12 min) + Kinesio-tape (changed every 2d) G3: Placebo (Laser—5 times/wk × 3 wks × 12 min)	VAS (pain), BCTQ, Pinch and grip strength, DML, DSL, SNCV, MA, SA.	0, 3, 12 weeks	G1: Improvement in VAS, BCTQ, pinch strength at 3 and 12 wks [05]; Grip strength at 3 wks [014]; MA and SNCV at 12 wks [05]. G2: Improvement in VAS, BCTQ, pinch and grip strength at 3 and 12 wks [05]; MA, DSL and SNCV at12 wks [05]. G3: Improvement in VAS and BCTQ (SSS) 3 wks [05]; DSL and SNCV at 12 wks [05]. G1 vs. G2: Greatest improvement in pinch and grip strength at 12 wks [<03] in G2. G1 vs. G3: Greatest Improvement in VAS and BCTQ at 3 and 12 wks [<01]; Grip strength at 3 wks [028] in G1. G2 vs. G3: All parameters at all times [<03] in G2.
**Hadianfard** **(2015)**	50 (3/47)	Mildand moderate. Idiopathic	G1: Acupuncture (2 times/wk × 4 wks) + Night splint G2: Control (Night splint+ Ibuprofen–3 times/day × 10 d)	BCTQ, VAS (pain), DSL, DML, SNCV.	0, 4 weeks	G1: Improvement in VAS, BCTQ, DSL, DML, SNCV [<001] G2: Improvement in VAS, BCTQ, DSL, DML, SNCV [<001] G1 vs. G2: Improvement in VAS [<001], BCTQ [<001], DSL [<001], SNCV [002]
**Incebiyik** **(2015)**	52 hands (0/28)	Mild and moderate. Idiopathic	G1: Short wave diathermy (15 min × 5 times/wk × 3 wks) + Gliding exercises G2: Placebo (diathermy off–15 min × 5 times/wk × 3 wks) + Gliding exercises	VAS (pain), BCTQ, clinical parameters.	0, 3 weeks	G1: Improvement in all variables [<001] G2: No improvement. G1 vs. G2: Improvement in all variables [<05] in G1.
**Karimzadeh** **(2019)**	73 (23/50)	Mild and moderate. Idiopathic	G1: 20 mg Triamcinolone G2: 40 mg Triamcinolone G3: 20 mg Methylprednisolone G4: 40 mg Methylprednisolone	VAS (pain), BCTQ, Grip strength, CMAP, SNAP, MNCV.	0, 3 months	G1: Improvement in all variables [<026] except CMAP. G2: Improvement in all variables [<034]. G3: Improvement in all variables [<017] except grip strength y CMAP. G4: Improvements in all variables [<01] except CMAP. G1 vs. G2 vs. G3 vs. G4: Significant difference only in amount in BCTQ-FSS [005] at 40 mg.
**Kumnerddee** **(2010)**	60 (6/54)	Mild and moderate. Idiopathic	G1: Electro-acupuncture (2 times/wk × 5 wks) G2: Control (Night splint—5 wks)	BCTQ, VAS (pain)	0, 5 weeks	G1: Improvement in all variables [<05]. G2: Improvement in BCTQ-SSS [008]. G1 vs: G2: Significant improvement in VAS [028] in G1.
**Ordahan** **(2017)**	60 (0/60)	Mild and moderate. Idiopathic	G1: Paraffin (5 days/wk × 3 wks) + Splint (always for 3 wks) + Exercises (2 times/day) G2: Control (Splint (always for 3 wks) + Exercises (2 times/day))	BCTQ, VAS (pain), DML, DSL, SNAP, MNCV, CMAP.	0, 3 weeks	G1: Improvement in all variables [<03] except CMAP. G2: Improvement in VAS y BCTQ-SSS [<02]. G1 vs. G2: Significant improvement BCTQ-FSS [021]; SNAP [028]; MNCV [041] in G1.
**Raeissadat** **(2018)**	41 (0/41)	Mild and moderate. Idiopathic	G1: Leukocyte-poor platelet-rich plasma (1 injection) + Night splint (8 wks) G2: Control (Night splint—8 wks)	BCTQ, VAS (pain), SNAP, CMAP	0, 10 weeks	G1: Improvement in all variables [<01] except CMAP. G2: Improvement in all variables [<01]. G1 vs. G2: No significant differences.
**Senna** **(2019)**	85 (14/71)	Mild and moderate. Idiopathic	G1: Platelet-rich plasma (1 dose) G2: 1ml methylprednisolone acetate (1 dose)	BCTQ, VAS (pain and paraesthesia), Phalen, Tinel, DML, CMAP, SL, SNAP, CSA.	0, 1 and 3 months	G1: Improvement in all variables [<01] at all follow-ups. G2: Improvement in the variables [<01] at all follow-ups. G1 vs. G2: No significant differences a 1 m. Significant improvement to 3 m in VAS-pain [041]; VAS-paraesthesia [040]; Phalen [041]; Tinel [039], BCTQ-SSS [007]; BCTQ-SSS [002]; DML [002]; SL [037]; SC [049] in G1.
**Wang** **(2017)**	52 (41/11)	Mild and moderate. Idiopathic	G1: Triamcinolone + lidocaine (1 ultrasound-guided dose) + Splint (as often as possible for 12 wks) G2: Triamcinolone + lidocaine (1 ultrasound-guided dose)	BCTQ, VAS (pain), DML, SNCV, CMAP, SNAP.	0, 6, 12 weeks	G1: Improvement in all variables except CMAP [<001] 6 and 12 wks. G2: Improvement in all variables except CMAP [<05] 6 and 12 wks. G1 vs. G2: Significant improvement at 12 wks in BCTQ-SSS [0032], BCTQ-FSS [.019] SNCV [015], SNAP [025] in G1.
**Wolny** **(2019)**	103 (11/92)	Mild and moderate. Idiopathic	G1: Neurodynamic techniques (20 min × 2 times/wk × 10 wks) G2: Control	BCTQ, NPRS (pain), Pinch and grip strength, SNCV, MNCV, MT.	0, 10 weeks	G1 vs. G2: Significant improvement SNCV [<01], MT [<01], NPRS [<01], BCTQ-SSS [<01], BCTQ-FSS [<01] in G1.
**Wolny** **(2017)**	140 (18/122)	Mild and moderate. Idiopathic	G1: Neurodynamic techniques + Function massage + Joint mobilization (15 × 2 times/wk × 10 wks). G2: Laser + ultrasound (15 × 2 times/wk × 10 wks).	BCTQ, VAS (pain), SNCV, MNCV, MT, SL	0, 10 weeks	G1: Improvement in all variables [<01] G2: Improvement in all variables [<05] except SNCV and MNCV. G1 vs. G2: Significant improvement in VAS [<01], BCTQ-SSS [<01], BCTQ-FSS [<01] in G1.
**Wu** **(2018)**	54 (11/43)	Mild and moderate. Idiopathic	G1: 5mL Dextrose (1 dose) G2: 3mL Triamcinolone	VAS (pain), BCTQ, CSA, SNCV, DML.	0, 1, 3, 4, 6 months	G1: Improvement in all variables at all times [<03] except DML at 1 and 6 months. G2: Improvement in VAS, BCTQ-SSS, BCTQ-FSS n 1, 3, 4 m [<01] and VAS 6 m [<001] G1 vs. G2: Significant improvement VAS at 4 m [<01] and 6 m [<001]; BCTQ-SSS at 4 m [<01] and 6 m [<001] and BCTQ-FSS at 4 m and 6 m [<001] in G1.
**Wu** **(2017)**	60 (10/50)	Mild and moderate. Idiopathic.	G1: Perineural injection of 5% dextrose (1 dose) G2: Placebo (1 dose of perineural injection with saline)	VAS (pain), BCTQ, CSA, SNCV, DML.	0, 1, 3, 6 months	G1: Improvement in all variables at all times [<05] except DML. G2: Improvement in VAS, BCTQ-SSS, BCTQ-FSS (not at 1 month) and CSA at all times [<05] G1 vs. G2: Significant improvement in VAS 1m [<01], 3m [<05], 6m [<001]; BCTQ-SSS 1m [<05], 3m [<.05], 6m [<.001]; BCTQ-FSS 1 m [<001], 3 m [<001], 6 m [<001]; SNC 1 m [03], 3 m [001], 6 m [006]; DML 1 m [04], 3 m [03]; CSA 3 m [01], 6 m [004].
**Xu** **(2020)**	55 (9/46)	Mild and moderate. Idiopathic	G1: Radial extracorporeal shock waves (1 time/wk × 3 wks) G2: Betamethasone 1 mL (1 dose)	VAS (pain), BCTQ, SNAPa, SNAPdl, CMAPa, CMAPdl	0, 3, 9 and 12 weeks	G1: Improvement in VAS, BCTQ, SNAPdl and CMAPdl 12 wks [<05]; VAS, BCTQ and SNAPdl 9 wks [<05]; VAS and BCTQ 3 wks [<05]. G2: Improvement in VAS and BCTQ 3 wks [05]; BCTQ 9 wks [05]. G1 vs. G2: Significant improvement VAS 9 wks [<001], 12 wks [<001]; BCTQ 9 wks [<001], 12 wks [<001]; SNAPdl 12 wks [004] in G1.
**Yildiz** **(2011)**	44 (8/36)	Mild and moderate. Idiopathic	G1: Ultrasound + Gel with ketoprofen (15 min × 5 times/wk × 2 wks) + Splint (8 wks) G2: Ultrasound + Gel with drugs (15 min × 5 times/wk × 2 wks) + Splint (8 wks) G3: Placebo (ultrasound off + gel without drugs) (15 min × 5 times/wk × 2 wks) + Splint (8 wks)	VAS (pain), BCTQ	0, 2 and 8 weeks	G1: Improvement in all variables 2 and 8 wks. G2: Improvement in all variables 2 and 8 wks. G3: Improvement in all variables 2 and 8 wks. G1 vs. G2: Significant improvement in VAS 8 wks [004] in G1. G1 vs. G3: Significant improvement in VAS 8 wks [002] in G1. G2 vs. G3: No significant differences.

BCTQ: Boston Carpal Tunnel Questionnaire; BCTQ-SSS: BCTQ Symptom Severity Scale; BCTQ-FSS: BCTQ Function Severity Scale; CMAP: compound muscle action potential; CMAPa: CMAP amplitude; CMAPdl: CMAP distal latency; CSA: cross-sectional area; DASH: Disability of Arm, Shoulder and Hand questionnaire; DML: distal motor latency; DSL: distal sensitivity latency; EMG: electromyography; F: female; M: male; m: month(s); MA: motor amplitude; min: minutes; MNCV: motor nerve conduction velocity; MT: motor latency; NPRS: Numeric Pain Rating Scale; SA: sensory amplitude; SL: sensory latency; SNAP: sensory.nerve action potential; SNAPa: SNAP amplitude; SNAPdl: SNAP distal latency; SNCV: sensory nerve conduction velocity; TNDM: test of neurodynamic median nerve mobilization; VAS: visual analogue scale; wk(s): week(s).

**Table 3:** Characteristics of Studies: Studies Including Severe and Systemic Condition.

Study	Sample		Groups	Outcomes	Follow-ups	Results
	No. (M/F)	CTS Characteristics				
**Atthakomol** **(2018)**	25 (6/19)	Mild, moderate and severe. Idiopathic	G1: Radial extracorporeal shock waves (3–7 min + 15 min cold) G2: Local corticosteroid injections (triamcinolone, lidocaine)	BCTQ, VAS (pain), DSL, DML, SNAP, CMAP	0, 1, 4, 12 and 24 weeks	G1: Improvement in VAS at 12 and 24 wks [05], BCTQ at 4 [05], 12 and 24 wks [01]; DSL at 12 wks [01]. G2: Improvement in BCTQ at 1 and 4 wks [01]; DSL 12 wks [01]. G1 vs. G2: Improvement in BCQT at 12 a 24 wks [01] G1; DSL at 0 and 12 wks [01] G1.
**Chen** **(2015)**	36 (1/35)	Mild, moderate and severe. Idiopathic, hypertension (6) and diabetics (6)	G1: Pulsed radiofrequency (32 min) + Night splint (8 h) G2: Control (Night splint–8h)	VAS (pain), BCTQ, CSA, SNCV, Pinch strength	0, 1, 4, 8, 12 weeks	G1 vs. G2: All parameters following intervention [<05]. Greatest improvement in VAS and BCTQ at all times, except BCTQ-SSS, in G1. All parameters improved in both.
**Chung** **(2016)**	181 (158/23)	Mild, moderate and severe. Idiopathic.	G1: Electro-acupuncture (13 sessions × 20 min) + 8 h Night splint G2: Control (8 h Night splint)	BCTQ, DASH, NPRS (pain), Sensitivity, Manual dexterity, Pinch strength.	0, 1, 2, 5, 17 weeks	G1 vs. G2: Improvement in BCTQ-FSS and DASH [05] at 1 and 17 wks. Improvement in manual dexterity at 17 wks [<01].
**Hamzeh** **(2020)**	52 hands (4/37)	Mild, moderate and severe. Idiopathic	G1: Neurodynamic technique with exercises to do at home (1 h × 1 time/wk × 4 wks) G2: Exercise programme (1h × 1time/wk × 4 wks supervision, 2 v/d)	BCTQ, Quick DASH, NPRS (pain), RDM, Grip strength.	0, 1, 6 months	G1: Improvement in BCTQ, NPRS, DASH, RDM, grip strength [<05] G2: Improvement in BCTQ, NPRS, DASH, RDM [<05] G1 vs. G2: Improvement in BCTQ-FSS [.004], DASH [004] in G1.
**Horng** **(2011)**	53 (3/50)	Mild, moderate and severe. Idiopathic	G1: Tendon gliding exercises (3 times/day) + Paraffin (2 times/wk) + Night splint (8 wks) G2: Nerve gliding exercises (3 times/day) + Paraffin (2 times/wk) + Night splint (8 wks) G3: Paraffin (2 v/wk) + Night splint (8 wks)	BCTQ, VAS (pain), DASH, WHOQOL (4 domains).	0, 8 weeks	G1: Improvement in BCTQ, VAS, DASH, WHOQOL (2 domains) [05]. G2: Improvement in BCTQ-SSS, VAS, WHOQOL (1 domain) [05]. G3: Improvement in BCTQ-SSS, VAS, WHOQOL (1 domain) [05]. G1 vs. G2 vs. G3: Significant improvement in BCTQ-FSS in G1 [04]
**Talebi** **(2018)**	30 (–/–)	Mild, moderate and severe. Diabetic	G1: Manual therapy–Nerve + interface mobilization + neural (25 min × 3 times/wk × 4 wks) G2: TENS (20 min) + Ultrasound (5 min) (3 times/wk × 4 wks)	VAS (pain), BCTQ, TNDM test.	0, 4 weeks	G1: Improvement in all variables [<009] G2: Improvement in VAS and BCTQ-SSS [<001] G1 vs. G2: Significant improvement in BCTQ-SSS [006]; BCTQ-FSS [043]; TNDM [<001] in G1
**Tezel** **(2019)**	44 (2/42)	Mild, moderate and severe. Idiopathic	G1: Acupuncture (2 times/wk x 5 wks) + Night splint G2: Control (Night splint)	BCTQ, NHP (6 QoL domains), VAS (pain), DML, MNCV, SCV, DSL, SNCV.	0, 5 weeks	G1 vs. G2: Significant improvement in VAS [007] and NHP-Pain [001] in G1.
**Wu** **(2016)**	40 (5/35)	Mild, moderate and severe. Idiopathic, hypertension (8) and diabetics (4)	G1: Radial extracorporeal shock waves + Splint G2: Placebo (shock waves) + Splint	VAS (pain, paraesthesia), BCTQ, CSA, SNCV, Pinch strength.	1, 4, 8 and 12 weeks	G1: Improvement in all variables at all times [<01] except SNCV 1 wk. G2: Improvement in all variables at all times [<05] except BCTQ-FSS 8 wks and 12 wks. G1 vs. G2: Significant improvement in VAS at 1 wk [<001], 4 wks [<001], 8 wks [003], 12 wks [006]; BCTQ-SSS at 1 wk [017], 4 wks [005], 8 wks [008]; BCTQ-FSS at 1 wk [001], 4 wks [002], 8 wks [002], 12 wks [007]; CSA at 1 wk [07], 8 wks [041], 12 wks [043] in G1.

BCTQ: Boston Carpal Tunnel Questionnaire; BCTQ-SSS: BCTQ Symptom Severity Scale; BCTQ-FSS: BCTQ Function Severity Scale; CMAP: compound muscle action potential; CMAPa: CMAP amplitude; CMAPdl: CMAP distal latency; CSA: cross-sectional area; DASH: Disability of Arm, Shoulder and Hand questionnaire; DML: distal motor latency; DSL: distal sensitivity latency; EMG: electromyography; F: female; M: male; m: month(s); MA: motor amplitude; min: minutes; MNCV: motor nerve conduction velocity; MT: motor latency; NPRS: Numeric Pain Rating Scale; SA: sensory amplitude; SL: sensory latency; SNAP: sensory nerve action potential; SNAPa: SNAP amplitude; SNAPdl: SNAP distal latency; SNCV: sensory nerve conduction velocity; TNDM: test of neurodynamic median nerve mobilization; VAS: visual analogue scale; WHOQOL: Word Health Organization (WHO) Quality Of Life scale; wk(s): week(s).

**Table 4:** Pain and BCTQ Results of Studies.

Group	Study	Treatment	Outcome Pain				Outcome BCTQ					
							SSS			FSS		
**Pharmacological**	**Karimzadeh (2019)**	G1. 20 mg Triamcinolone G2. 40 mg Triamcinolone G3. 20 mg Methylprednisolone G4. 40 mg Methylprednisolone	VAS G1. Pre (5.4 ± 2.4) vs. Post (4 ± 1.8) *p* = 0.007 G2. Pre (5.4 ± 2.4) vs. Post (3.7 ± 2.2) *p* = 0.001 G3. Pre (5.6 ± 1.8) vs. Post (4.1 ± 1.9) *p* = 0.014 G4. Pre (5.3 ± 2.9) vs. Post (3 ± 2.2) *p* = 0.006			Triamcinolone vs. Methylprednisolone 3.84 ± 2.1 vs. 3.53 ± 2.1 (*p* = 0.53) 20 mg vs. 40 mg 4.05 ± 1.8 vs. 3.36 ± 2.2 (*p* = 0.14)	G1. Pre (32.5 ± 11.8) vs. Post (24.9 ± 10.2) *p* = 0.002 G2. Pre (31.7 ± 8.6) vs. Post (22.3 ± 1.1) *p* = 0.001 G3. Pre (31.9 ± 8) vs. Post (.1 ± 1.9) *p* = 0.001 G4. Pre (5.3 ± 2.9) vs. Post (3 ± 2.2) *p* = 0.001 Triamcinolone vs. Methylprednisolone 23.5 ± 10.5 vs. 22.6 ± 7.7 (*p* = 0.67) 20 mg vs. 40 mg 23.88 ± 9.3 vs. 22.44 ± 9.1 (*p* = 0.50)			G1. Pre (32.5 ± 11.8) vs. Post (24.9 ± 10.2) *p* = 0.002 G2. Pre (31.7 ± 8.6) vs. Post (22.3 ± 1.1) *p* = 0.001 G3. Pre (31.9 ± 8) vs. Post (.1 ± 1.9) *p* = 0.001 G4. Pre (5.3 ± 2.9) vs. Post (3 ± 2.2) *p* = 0.002 Triamcinolone vs. Methylprednisolone 14.0 ± 4.9 vs. 15.3 ± 5.3 (*p* = 0.27) 20 mg vs. 40 mg 16.46 ± 5.7 vs. 13.05 ± 4.4 (*p* =0.005)		
	**Raeissadat (2018)**	G1. Leukocyte-poor platelet-rich plasma + Night splint G2. Night splint	VAS G1. Pre (6.82 ± 1.24) vs. Post (4.02 ± 1.92) *p* < 0.001 G2. Pre (6.24 ± 1.13) vs. Post (3.52 ± 2.02) *p* < 0.001 G1 vs. G2–2.90 ± 2.1 vs. -2.76 ± 2.4 (*p* = 0.845; Power = 0.073; *p* = 0.098 adjusted for age).				G1. Pre (2.43 ± 0.73) vs. Post (17 ± 0.52) *p* < 0.001 G2. Pre (2.76 ± 0.40) vs. Post (1.90 ± 0.42) *p* < 0.001 G1 vs. G2. -0.05 ± 0.2 vs. −0.19 ± 0.4 (*p* = 0.174; Power = 0.393; *p* = 0.194 adjusted for age).			G1. Pre (2.54 ± 0.63) vs. Post (1.82 ± 0.42) *p* < 0.001 G2. Pre (2.36 ± 0.83) vs. Post (1.83 ± 0.73) *p* < 0.001 G1 vs. G2 −0.86 ± 0.5 vs. −0.63 ± 0.8 (*p* = 0.289; Power = 0.283; *p* = 0.554 adjusted for age).		
	**Senna (2019)**	G1. Platelet-rich plasma G2: 1 mL methylprednisolone acetate	VAS G1 (F = 150,217; *p* < 0.001) Pre: 68.1 ± 6.0 1 month: 24.4 ± 7.3 3 months: 21.8 ± 6.5	G2 (F = 116,217; *p* < 0.001) Pre: 69.5 ± 4.9 1 month: 25.9 ± 8.3 3 months: 25.2 ± 7.1		G1 vs. G2 Pre: t = 1.178; *p* = 0.242 1 month: t = 0.337; *p* = 0.737 3 months: t = 2.100; *p* = 0.040	G1 (F= 94,739; *p* < 0.001) Pre: 3.5 ± 0.4 1 month: 2.4 ± 0.6 3 months: 2.0 ± 0.7		G2 (F = 89,111; *p* < 0.001) Pre: 3.4 ± 0.4 1 month: 2.5 ± 0.5 3 months: 2.4 ± 0.7	G1 (F=111,916; *p* < 0.001) Pre: 3.5 ± 0.4 1 month: 2.5 ± 0.5 3 months: 2.1 ± 0.6	G2 (F = 71,821; *p* < 0.001) Pre: 3.4 ± 0.5 1 month: 3.0 ± 0.4 3 months: 2.5 ± 0.6	
							G1 vs. G2 Pre: t = 1.1082; *p* = 0.274 1 month: t = 0.268; *p* = 0.790 3 months: t = 2.752; *p* = 0.007			G1 vs. G2 Pre: t = 1.282; *p* = 0.204 1 month: t = 1.283; *p* = 0.203 3 months: t = 1.385; *p* = 0.002		
	**Wang (2017)**	G1. Triamcinolone + lidocaine + Splint G2. Triamcinolone + lidocaine	VAS. G1 Pre: 3.61 ± 3.27 6 weeks: 0.80 ± 2.00 12 weeks: 1.00 ± 2.05	G2 Pre: 2.23 ± 2.59 6 weeks: 0.40 ± 0.84 12 weeks: 0.80 ± 1.57		G1 vs. G2 6 weeks: 0.40 (–0.45 to 1.25) *p* = 0.993 12 weeks: 0.19 (−0.82 to 1.21) *p* = 0.898	G1 Pre: 2.27 ± 0.71 6 weeks: 1.30 ± 0.53 12 weeks: 1.32 ± 0.43		G2 Pre: 1.96 ± 0.62 6 weeks: 1.28 ± 0.21 12 weeks: 1.49 ± 0.51	G1 Pre: 1.93 ± 0.78 6 weeks: 1.33 ± 0.56 12 weeks: 1.27 ± 0.50	G2 Pre: 1.61 ± 0.62 6 weeks: 1.18 ± 0.28 12 weeks: 1.32 ± 0.35	
							G1 vs. G2 6 weeks: 0.02 (−0.20 to 0.24) *p* = 0.216 12 weeks: -0.17 (-0.43 to 0.09) *p* = 0.246			G1 vs. G2 6 weeks: 0.14 (−0.10 to 0.40) *p* = 0.654 12 weeks: -0.04 (-0.29 to 0.19) *p* = 0.134		
	**Wu (2017)**	G1: Perineural injection of 5% dextrose G2: Perineural injection with saline	VAS. G1 Pre: 6.67 ± 0.30 1 month: 4.60 ± 0.35 (*p* < 0.001) 3 months: 3.57 ± 0.30 (*p* < 0.001) 6 months: 2.43 ± 0.30 (*p* < 0.001)	G2 Pre: 6.56 ± 0.30 1 month: 5.64 ± 0.35 (*p* = 0.002) 3 months: 4.70 ± 0.46 (*p* < 0.001) 6 months: 4.59 ± 0.46 (*p* < 0.001)		G1 vs. G2 1 month: −2.07 ± 0.24 vs. −0.93 ± 0.21 (*p* = 0.001) 3 months: −3.10 ± 0.35 vs. −1.86 ± 0.37 (*p* = 02) 6 months: −4.23 ± 0.33 vs. −1.98 ± 0.37 (*p* < 0.001)	G1 Pre: 30.20 ± 1.25 1 month: 20.83 ± 1.06 (*p* < 0.001) 3 months: 17.60 ± 0.80 (*p* < 0.001) 6 months: 15.30 ± 0.60 (*p* < 0.001)		G2 Pre: 28.07 ± 1.93 1 month: 22.37 ± 1.76 (*p* <.001) 3 months: 20.50 ± 2.02 (*p* < 0.001) 6 months: 21.60 ± 2.06 (*p* = 0.002)	G1 Pre: 21.87 ± 0.69 1 month: 14.17 ± 0.72 (*p* < 0.001) 3 months: 12.90 ± 0.52 (*p* < 0.001) 6 months: 11.43 ± 0.46 (*p* < 0.001)	G2 Pre: 19.93 ± 0.96 1 month: 18.00 ± 1.05 (*p* = 09) 3 months: 16.77 ± 1.18 (*p* = 0.005) 6 months: 17.07 ± 1.23 (*p* = 03)	
							G1 vs. G2 1 month: −9.37 ± 1.20 vs. -5.70 ± 0.93 (*p* = 0.02) 3 months: −12.60 ± 1.19 vs. -7.57 ± 1.54 (*p* = 0.01) 6 months: -14.90 ± 1.24 vs. −6.47 ± 1.46 (*p* <0.001)			G1 vs. G2 1 month: −7.70 ± 0.97 vs. −1.93 ± 0.65 (*p* < 0.001) 3 months: −8.97 ± 0.73 vs. −3.17 ± 0.79 (*p* < 0.001) 6 months: −10.43 ± 0.83 vs. -2.87 ± 0.86 (*p* < 0.001)		
	**Wu (2018)**	G1. 5mL Dextrose G2. 3mL Triamcinolone	VAS. G1 Pre: 6.3 ± 0.3 1 month: 4.2 ± 0.3; −2.1 (−1.4 to −2.8) (*p* < 0.001) 3 months: 3.3 ± 0.2 −3.1 (−2.2 to −3.9) (*p* < 0.001) 4 months: 2.8 ± 0.3 −3.6 (−2.6 to −4.5) (*p* < 0.001) 6 months: 2.0 ± 0.3 −4.3 (−3.2 to −5.4) (*p* < 0.001)	G2 Pre: 6.2 ± 0.2 1 month: 4.2 ± 0.4 −2.1 (−1.0 to −3.2) (*p* ≤ 0.001) 3 months: 3.57 ± 0.30 −2.6 (−1.7 to −3.5) (*p* < 0.001) 4 months: 3.57 ± 0.30 −2.3 (−1.4 to −3.3) (*p* < 0.001) 6 months: 2.43 ± 0.30 −1.7 (−0.7 to −2.7) (*p* < 0.001)		G1 vs. G2 1 month: (*p* > 05) 3 months: (*p* > 05) 4 months (*p* < 01) 6 months (*p* > 001)	G1 Pre: 28.2 ± 1.2 1 month: 19.8 ± 0.9; −8.4 (−4.5 to −12.4) (*p* < 0.001) 3 months: 16.4 ± 0.7; −3.1 (−2.2 to −3.9) (*p* < 0.001) 4 months: 15.9 ± 0.6; −12.3 (−8.4 to −16.2) (*p* < 0.001) 6 months: 14.7 ± 0.6; −13.5 (−9.3 to −17.6) (*p* < 0.001)		G2 Pre: 27.6 ± 1.4 1 month: 22.5 ± 1.7; −5.0 (−0.6 to −9.4) (*p* = 016) 3 months: 19.8 ± 1.2; −7.8 (−3.5 to −12.0) (*p* < 0.001) 4 months: 21.2 ± 1.3; −6.4 (−1.8 to −10.9) (*p* = 0.002) 6 months: 23.7 ± 1.6; −3.9 (0.6 to −8.3) (*p* = 128)	G1 Pre: 20.7 ± 1.1 1 month: 15.0 ± 0.8; −5.7 (−2.6 to −8.9) (*p* < 0.001) 3 months: 3.3 ± 0.2; −3.1 (−2.2 to −3.9) (*p* < 0.001) 4 months: 2.8 ± 0.3; −3.6 (−2.6 to −4.5) (*p* < 0.001) 6 months: 2.0 ± 0.3; −4.3 (−3.2 to −5.4) (*p* < 0.001)	G2 Pre: 19.7 ± 0.8 1 month: 16.1 ± 1.0; −3.6 (−0.7 to −6.5) (*p* < 0.001) 3 months: 15.0 ± 0.8; −4.7 (−2.1 to −7.2) (*p* < 0.001) 4 months: 15.9 ± 0.8; −3.7 (−1.1 to −6.4) (*p* < 0.001) 6 months: 16.6 ± 0.8; −3.0 (1.0 to −6.2) (*p* = 063)	
							G1 vs. G2 1 month: (*p* > 05) 3 months: (*p* > 05) 4 months: (*p* < 01) 6 months: (*p* < 0.001)			G1 vs. G2 1 month: (*p* > 05) 3 months: (*p* > 05) 4 months: (*p* < 0.001) 6 months: (*p* < 0.001)		
**Electrotherapy**	**Çatalbas (2018)**	G1. Continuous ultrasound + Night splint G2. Pulsed ultrasound + Night splint G3. Placebo Ultrasound + Night splint	VAS. G1 Pre: 5.5 ± 3.2 2 weeks: 2.8 ± 1.9 (*p* < 0167) 6 weeks: 2.0 ± 2.2 (*p* < 0167) vs. pre and post.	G2 Pre: 5.4 ± 2.5 2 weeks: 2.9 ± 2.4 (*p* < 0167) 6 weeks: 2.1 ± 2.3 (*p* < 0167) vs. pre.		G3 Pre: 5.0 ± 2.5 2 weeks: 2.6 ± 2.1 (*p* < 0167) 6 weeks: 2.2 ± 2.0 (*p* < 0167) vs. pre.	G1 Pre: 2.8 ± 0.8 2 weeks: 1.9 ± 0.7 (*p* < 0167) 6 weeks: 1.9 ± 0.7 (*p* < 0167) vs. pre.	G2 Pre: 3.1 ± 0.8 2 weeks: 2.1 ± 0.7 (*p* < 0167) 6 weeks: 2.1 ± 0.9 (*p* < 0167) vs. pre.	G3 Pre: 2.8 ± 0.9 2 weeks: 2.2 ± 0.8 (*p* < 0167) 6 weeks: 2.2 ± 0.7 (*p* < 0167) vs. pre.	G1 Pre: 2.5 ± 0.9 2 weeks: 1.8 ± 0.7 (*p* < 0167) 6 weeks: 2.4 ± 0.8 (*p* < 0167) vs. pre.	G2 Pre: 2.4 ± 0.9 2 weeks: 1.9 ± 0.8 (*p* < 0167) 6 weeks: 1.9 ± 0.8 (*p* < 0167) vs. pre.	G3 Pre: 2.4 ± 0.8 2 weeks: 1.9 ± 0.6 (*p* < 0167) 6 weeks: 1.9 ± 0.7 (*p* < 0167) vs. pre.
			Pre: *p* = 0.730 2 weeks: *p* = 0.860 6 weeks: *p* = 0.850				Pre: *p* = 0.380; 2 weeks: *p* = 0.600; 6 weeks: *p* = 0.430			Pre: *p* = 0.910; 2 weeks: *p* = 0.580; 6 weeks: *p* = 0.260		
	**Chen (2015)**	G1. Pulsed radiofrequency + Night splint G2. Night splint	VAS. G1 Pre: 5.4 ± 2.1 1 week: 2.5 ± 1.4 −2.9 ± 1.1 (2.4–3.4). 4 weeks: 2.2 ± 1.5 −3.2 ± 1.1 (2.6–3.7). 8 week: 1.8 ± 1.2 −3.6 ± 1.6 (2.8–4.4). 12 weeks: 1.1 ± 0.8 −4.2 ± 2.1 (3.2–5.2).	G2 Pre: 5.0 ± 1.5 1 week: 3.9 ± 1.1 −1.1 ± 1.2 (0.5–1.7). 4 weeks: 3.4 ± 1. −1.6 ± 1.4 (0.9–2.2) 8 week: 3.2 ± 1.1 −1.8 ± 1.5 (1.0–2.5) 12 weeks: -2.0 ± 1.8 (1.1–2.9)		G1 vs. G2 Pre: (*p* = 552) 1 week: (*p* = 0.002) 4 weeks: (*p* = 013) 8 week: (*p* < 0.001) 12 weeks: (*p* < 0.001)	G1 Pre: 33.4 ± 6.4 1 week: 21.7 ± 7.3 −11.7 ± 4.7 (9.4–14.1). 4 weeks: 19.6 ± 4.8 −13.9 ± 5.1 (11.3–16.4). 8 week: 17.1 ± 3.6 −16.3 ± 6.3 (13.2–19.4). 12 weeks: 13.7 ± 2.1 −19.7 ± 6.7 (16.4–23.0).		G2 Pre: 33.0 ± 6.5 1 week: 26.0 ± 6.7 −7.0 ± 6.6 (3.7–10.3) 4 weeks: 23.6 ± 6.2 −9.4 ± 7.7 (5.6–13.3) 8 week: 22.8 ± 6.7 −10.2 ± 8.7 (5.9–14.5) 12 weeks: 22.1 ± 6.3 −10.9 ± 9.2 (6.3–15.5)	G1 Pre: 23.3 ± 3.4 1 week: 12.1 ± 3.9 −11.2 ± 4.6 (8.9–13.4) 4 weeks: 11.2 ± 3.8 −12.1 ± 4.3(10–14.3) 8 week: 10.7 ± 3.7 −12.6 ± 5(10.1–15.1). 12 weeks: 9.1 ± 2.2 −14.2 ± 4.1(12.2–16.3).	G2 Pre: 23.2 ± 6.2 1 week: 16.3 ± 6.9 −6.9 ± 6.3 (3.8–10) 4 weeks: 14.6 ± 4.9 −8.6 ± 6.8 (5.2–11.9) 8 week: 14.4 ± 5.0 −8.8 ± 7.0 (5.3–12.2) 12 weeks: 13.9 ± 5.0 −9.3 ± 7.4 (5.6–13.0)	
							G1 vs. G2 Pre: (*p* = 837) 1 week: (*p* = 077) 4 weeks: (*p* = 037) 8 week: (*p* = 0.004) 12 weeks: (*p* < 0.001)			G1 vs. G2 Pre: (*p* = 948) 1 week: (*p* = 032) 4 weeks: (*p* = 025) 8 week: (*p* = 016) 12 weeks: (*p* = 0.001)		
	**Chung (2016)**	G1. Electro-acupuncture + Night splint G2. Night splint	NPRS. G1 Pre: 4.38 ± 2.62 1 week: 18 (20.0) –0.22 (–0.68 to 0.23) 2 weeks: 21 (23.3) –0.30 (–0.81 to 0.21) 5 week: 27 (31.0) –0.68 (–1.18 to –0.19) 17 weeks: 34 (40.0) –1.22 (–1.79 to –0.65)	G2 Pre: 4.52 ± 2.78 1 week: 20 (22.0) –0.43 (–0.89 to 0.04) 2 weeks: 24 (26.4) –0.50 (–1.01 to 0.01) 5 week: 28 (31.1) −0.55 (–1.11 to 0.02) 17 weeks: 31 (34.8) –0.61 (–1.22 to 0.00)		G1 vs. G2 1 week: (*p* = 6) 4 weeks: (*p* = 7) 8 week: (*p* = 6) 12 weeks: (*p* = 03)	G1 Pre: 2.32 ± 0.62 1 week: 11 (12.2) 0.04 (–0.03 to 0.12) 2 weeks: 19 (21.1) –0.01 (–0.09 to 0.07) 5 week: 33 (37.9) 0.17 (–0.28 to –0.06) 17 weeks: 40 (47.1) –0.25 (–0.37 to –0.12)		G2 Pre: 2.40 ± 0.69 1 week: 19 (20.9) 0.01 (–0.08 to 0.10) 2 weeks: 23 (25.3) –0.02 (–0.13 to 0.08) 5 week: 27 (30.0) –0.06 (–0.19 to 0.07) 17 weeks: 32 (36.0) –0.09 (–0.25 to 0.06)	G1 Pre: 4.38 ± 2.62 1 week: 7 (7.8) 0.14 (0.05 to 0.23) 2 weeks: 15 (16.7) 0.11 (0.00 to 0.22) 5 week: 19 (21.8) –0.01 (–0.12 to 0.11) 17 weeks: 30 (35.3) –0.16 (–0.28 to –0.04)	G2 Pre: 4.52 ± 2.78 1 week: 17 (18.7) 0.09 (0.00 to 0.18) 2 weeks: 15 (16.5) 0.07 (–0.04 to 0.17) 5 week: 18 (20.0) 0.06 (–0.07 to 0.18) 17 weeks: 21 (23.6) 0.02 (–0.13 to 0.17)	
							G1 vs. G2 1 week: 0.02 (–0.09 to 0.13) (*p* = 8) 4 weeks: –0.01 (–0.13 to 0.11) (*p* = 9) 8 week:–0.15 (–0.29 to –0.01) (*p* = 0.04) 12 weeks: –0.20 (–0.36 to –0.03) (*p* = 02)			G1 vs. G2 1 week: 0.05 (–0.08 to 0.17) (*p* = 03) 4 weeks: 0.03 (–0.12 to 0.17) (*p* = 1.0) 8 week: –0.09 (–0.24 to 0.06) (*p* = 8) 12 weeks: –0.22 (–0.38 to –0.05) (*p* = 09)		
	**Fusakul (2014)**	G1. Diode laser + Splint G2. Laser Placebo + Splint	VAS. G1 Pre: 6.26 ± 0.27 (*p* < 0.05) 5 weeks: 4.25 ± 0.34 (*p* < 0.05) 12 weeks: 3.45 ± 0.38 (*p* < 0.05)	G2 Pre: 4.83 ± 0.33 (*p* < 0.05) 5 weeks: 3.15 ± 0.30 (*p* < 0.05) 12 weeks: 2.48 ± 0.36 (*p* < 0.05)		G1 vs. G2 Pre: *p* = 0.174 5 weeks: *p* = 0.243 12 weeks: *p* = 0.433	G1 Pre: 2.10 ± 0.68 (*p* < 0.05) 5 weeks: 1.68 ± 0.66 (*p* < 0.05) 12 weeks: 1.49 ± 0.58 (*p* < 0.05)		G2 Pre: 1.68 ± 0.56 (*p* < 0.05) 5 weeks: 1.43 ± 0.49 (*p* < 0.05) 12 weeks: 1.35 ± 0.51 (*p* < 0.05)	G1 Pre: 2.07 ± 0.67 (*p* < 0.05) 5 weeks: 1.75 ± 0.62 (*p* < 0.05) 12 weeks: 1.53 ± 0.57 (*p* < 0.05)	G2 Pre: 1.77 ± 0.62 (*p* < 0.05) 5 weeks: 1.54 ± 0.62 (*p* < 0.05) 12 weeks: 1.37 ± 0.49 (*p* < 0.05)	
							G1 vs. G2 Pre: *p* = 0.291 5 weeks: *p* = 0.031 12 weeks *p* = 0.886			G1 vs. G2 Pre: *p* = 0.712 5 weeks: *p* = 0.406 12 weeks: *p* = 0.313		
	**Güner (2018)**	G1. Low-intensity laser G2. Low intensity laser + Kinesio-tape G3. Laser Placebo	VAS daytime G1 Post: 6 (3–9) 3 weeks: 1 (0–5) *p* < 0.001 12 weeks:1 (0–5) *p* < 0.001	G2 Post: 6 (4–10) 3 weeks: 1 (0–8) *p* < 0.001 12 weeks:2 (0–6) *p* < 0.001	G3 Post: 6 (2–10) 3 weeks: 5 (1–9) *p* < 0.001 12 weeks:5 (0–9) *p* = 085	G1 vs. G2 0–3 w: *p* = 879 0–12w: *p* = 879 G1 vs. G3 0–3 w: *p* = 0.001 0-12w: *p* < 0.001 G2 vs. G3 0-3w: *p* = 0.003 0-12w: *p* < 0.001	G1 Post: 3.09 (2.09–4) 3 weeks: 1.36 (1.00–3.27) *p* < 0.001 12 weeks: 1.90 (1.00–3.00) *p* < 0.001	G2 Post: 3.24 (2.63–4.27) 3 weeks: 1.54 (1–2.81) *p* < 0.001 12 weeks: 1.90 (1–2.90) *p* < 0.001	G3 Post: 3.27 (2.09–4.18) 3 weeks: 2.63 (1.54–3.54) *p* = 0.001 12 weeks: 3.00 (1.36–3.81) *p* = 054	G1 Post: 2.63 (1.25–3.75) 3 weeks: 1.63 (1.00–3.25) *p* < 0.001 12 weeks: 1.63 (1.00–3.25) *p* < 0.001	G2 Post: 2.88 (1.38–3.88) 3 weeks: 1.63 (1.25–2.75) *p* < 0.001 12 weeks: 1.88 (1–3) *p* < 0.001	G3 Post: 3.12 (1.13–5.75) 3 weeks: 3 (1–5.75) *p* = 632 12 weeks: 2.5 (1–4.13) *p* = 626
			VAS night G1 Post: 8 (4–10) 3 weeks: 2 (0–8) *p* < 0.001 12 weeks:0 (0–8) *p* < 0.001	G2 Post: 8 (3–10) 3 weeks: 0 (0–8) *p* < 0.001 12 weeks:0 (0–6) *p* < 0.001	G3 Post: 7 (5–10) 3 weeks:5 (1–10) *p* = 0.001 12 weeks: 7 (0–10) *p* = 273	G1 vs. G2 0–3w: *p* = 245 0–12w: *p* = 577 G1 vs. G3 0–3w: *p* = 0.004 0–12w: *p* < 0.001 G2 vs. G3 0–3w: *p* = 0.001 0–12w: *p* < 0.001	G1 vs. G2 0–3w: *p* = 840 0–12w: *p* = 659	G1 vs. G3 0–3w: *p* = 0.002 0–12w: *p* = 0.006	G2 vs. G3 0–3w: *p* = 020 0–12w: *p* < 0.001	G1 vs. G2 0–3w: *p* = 772 0–12w: *p* = 970	G1 vs. G3 0–3w: *p* = 0.001 0–12w: *p* = 0.006	G2 vs. G3 0–3w: *p* = 0.002 0–12w: *p* = 0.007
	**Kumnerddee (2010)**	G1. Electro-acupuncture G2. Night splint	VAS. G1 Post vs. Post: 22.57 ± 22.27 vs. 7.97 + 14.99	G2 Post vs. Post: 22.57 ± 22.27 vs. 17.60 + 22.37		G1 vs. G2 9.63 (1.07 to 18.20) *p* = 0.028	G1 Post vs. Post: 2.03 ± 0.61 vs. 1.98 ± 0.56		G2 Post vs. Post: 0.39 vs. 1.66 ± 0.50	G1 Post vs. Post: 1.76 ± 0.63 vs. 1.50 ± 0.39	G2 Post vs. Post: 1.70 ± 0.57 vs. 1.54 ± 0.48	
							G1 vs. G2 0.11 (−0.10 to 0.33). *p* = 0.295			G1 vs. G2 0.05 (−0.16 to 0.25). *p* = 0.663		
	**Wu (2016)**	G1. Radial extracorporeal shock waves + Splint G2. Placebo shock waves + Splint	VAS. G1 Pre: 6.26 ± 0.27 1 week: 3.15 ± 0.98 *p* < 0.01) 4 weeks: 2.48 ± 1.00 (*p* < 0.01) 8 weeks: 2.77 ± 1.37 (*p* < 0.01) 12 weeks: 2.70 ± 1.23 (*p* < 0.01)	G2 Pre: 5.90 ± 1.22 1 week: 4.47 ± 1.05 (*p* < 0.05) 4 weeks: 4.12 ± 1.14 (*p* < 0.05) 8 weeks: 3.80 ± 1.35 (*p* < 0.05) 12 weeks: 3.59 ± 1.27 (*p* < 0.05)		G1 vs. G2 1 week: *p* < 0.001 4 weeks: *p* < 0.001 8 weeks: *p* = 0.003 12 weeks: *p* = 0.006	G1 Pre: 32.65 ± 7.86 1 week: 20.20 ± 5.0 (*p* < 0.001) 4 weeks: 18.90 ± 4.76 (*p* < 0.001) 8 weeks: 17.50 ± 4.11 (*p* < 0.001) 12 weeks: 18.45 ± 4.76 (*p* < 0.001)		G2 Pre: 29.95 ± 8.46 1 week: 23.75 ± 6.76 (*p* = 0.005) 4 weeks: 23.45 ± 7.01 (*p* = 0.009) 8 weeks: 22.00 ± 6.32 (*p* = 0.002) 12 weeks: 19.80 ± 5.04 (*p* = 0.002)	G1 Pre: 17.70 ± 4.21 1 week: 11.75 ± 2.51(*p* < 0.001) 4 weeks: 10.90 ± 2.81 (*p* < 0.001) 8 weeks: 10.70 ± 2.52 (*p* < 0.001) 12 weeks: 10.60 ± 2.28 (*p* < 0.001)	G2 Pre: 16.65 ± 5.03 1 week: 14.30 ± 4.46 (*p* = 0.021) 4 weeks: 13.70 ± 4.84 (*p* < 0.019) 8 weeks: 13.95 ± 5.48 (*p* = 0.109) 12 weeks: 13.50 ± 5.77 (*p* = 0.107)	
							G1 vs. G2 1 week: *p* = 0.017 4 weeks: *p* = 0.005 8 weeks: *p* = 0.008 12 weeks: *p* = 0.171			G1 vs. G2 1 week: *p*= 0.001 4 weeks: *p*= 0.002 8 weeks: *p*= 0.002 12 weeks: *p* = 0.007		
**Manual Therapy**	**Dinarvand (2017)**	G1. Unciform and scaphoid bone mobilization + Night splint G2. Night splint	VAS. G1 Post vs. Post: *p* < 0.001 5.44 ± 2.35 vs. 1.94 ± 1.34		G2 Post vs. Post: *p* < 0.001 6.36 ± 1.16 vs. 3.52 ± 2.06		G1 Post vs. Post: *p* < 0.001 2.58 ± 0.57 vs. 1.46 ± 0.37		G2 Post vs. Post: *p* < 0.001 6.36 ± 1.16 vs. 3.52 ± 2.06	G1 Post vs. Post: *p* < 0.001 2.52 ± 0.4 vs. 1.8 ± 0.44	G2 Post vs. Post: *p* < 0.001 2.61 ± 0.57 vs. 1.76 ± 0.45	
	**Geler-Külcu (2016)**	G1. Kinesio-tape + Gliding exercises G2. Placebo kinesio-tape + Gliding exercises G3. Night splint + Gliding exercises	VAS. G1 Post vs. Post: *p* = 0.005 6.6 ± 2.1 vs. 4.1 ± 2.7	G2 Post vs. Post: *p* = 0.003 5.8 ± 3.2 vs. 3.9 ± 2.8	G3 Post vs. Post: *p* = 0.024 6.1 ± 2.9 vs. 5.7 ± 3.1	G1 vs. G2 vs. G3 *p* = 0.269	G1 Post vs. Post: *p* < 0.0001 32 ± 8.4 vs. 20 ± 7.5	G2 Post vs. Post: *p* < 0.0001 33 ± 10.7 vs. 24.4 ± 8.0	G3 Post vs. Post: *p* < 0.036 31.6 ± 8.4 vs. 28.7 ± 11.8	G1 Post vs. Post: *p* = 0.001 23.1 ± 6.0 vs. 16.2 ± 5.4	G2 Post vs. Post: *p* = 0.077 19.7 ± 8.4 vs. 16.3 ± 5.8	G3 Post vs. Post: *p* = 0.090 19.7 ± 7.0 vs. 19.7 ± 19.7
							G1 vs. G2 vs. G3 *p* = 0.024			G1 vs. G2 vs. G3 *p* = 0.017		
	**Günay (2015)**	G1. Carpal bone mobilization + Splint G2. Splint	VAS daytime G1 Pre vs. Post: *p* = 0.003 3 (0–8) vs. 0 (0–8)	G2 Pre vs. Post: *p* = 0.011 5 (0–7) vs. 1 (0–7)		G1 vs. G2 −2 (−7;2) vs. -3 (−7;4): *p* = 0.53	G1 Pre vs. Post: *p* < 0.001 29 (20–46) vs. 17 (12–44)		G2 Pre vs. Post: *p* = 0.001 31.5 (18–46) vs. 23 (11–43)	G1 Pre vs. Post: *p* = 0.001 21 (14–33) vs. 16.5 (8–32)	G2 Pre vs. Post: *p* = 0.57 19 (9–35) vs. 19 (8–29)	
			VAS night G1 Pre vs. Post: *p* < 0.001 6 (1–8) vs. 0 (0–8)	G2 Pre vs. Post: *p* = 0.001 5 (0–9) vs. 0 (0–8)		G1 vs. G2 −5 (−8;2) vs. -4 (−8;2): *p* = 0.14	G1 vs. G2 −12.5 (−26;5) vs. −8.0 (−23;5): *p* = 0.39			G1 vs. G2 -5.5 (−18;2) vs. 0 (−11;5): *p* = 0.01		
	**Hadianfard (2015)**	G1. Acupuncture + Night splint G2. Night splint + Ibuprofen	VAS. G1 Post vs. Post: *p* < 0.001 7.32 ± 0.94 vs. 3.8 ± 0.78	G2 Post vs. Post: *p* < 0.001 7.32 ± 1.06 vs. 4.64 ± 0.7		G1 vs. G2 *p* = 0.001	G1 Post vs. Post: *p* < 0.001 6.6 ± 2.1 vs. 4.1 ± 2.7		G2 Post vs. Post: *p* < 0.001 6.6 ± 2.1 vs. 4.1 ± 2.7	G1 Post vs. Post: *p* < 0.001 17.708 ± 2.561 vs. 11.00 ± 0.780	G2 Post vs. Post: *p* < 0.001 18.00 ± 3.00 vs. 12.840 ± 1.929	
							G1 vs. G2 *p* < 0.001			G1 vs. G2 *p* < 0.001		
	**Hamzeh (2020)**	G1. Neurodynamic technique with home exercises G2. Exercise programme	NPRS mean G1 Pre: 4.17 ± 2.23 1 month: 1.22 ± 1.59 *p* < 05 6 months: 1.06 ± 1.75 *p* < 05	G2 Pre: 3.17 ± 2.49 1 month: 2.97 ± 2.44 *p* > 05 6 months: 2.09 ± 2.43 *p* > 05		G1 vs. G2 Pre: 1.00 (−0.3 to 2.3) *p* = 14 1 month: −1.75 (-2.9 to −0.6) *p* = 0.005 6 months: −1.03 (−2.5 to 0.4) *p* = 14	G1 Pre: 3.17 ± 0.86 1 month: 2.04 ± 0.68 *p* < 05 6 months: 1.64 ± 0.66 *p* < 05		G2 Pre: 2.71 ± 0.76 1 month: 2.16 ± 0.74 *p* < 05 6 months: 1.88 ± 0.60 *p* < 05	G1 Pre: 2.80 ± 0.87 1 month: 2.08 ± 0.82 *p* < 05 6 months: 1.35 ± 0.48 *p* < 05	G2 Pre: 2.63 ± 0.84 1 month: 2.17 ± 0.97 *p* < 05 6 months: 1.84 ± 0.87 *p* < 05	
			NPRS worst G1 Pre: 7.52 ± 2.57 1 month: 3.17 ± 2.59 *p* < 05 6 months: 2.88 ± 3.39 *p* < 05	G2 Pre: 7.07 ± 2.19 1 month: 5.10 ± 2.81 *p* < 05 6 months: 4.82 ± 2.81 *p* < 05		G1 vs. G2 Pre: 0.45 (−0.9 to 1.8) *p* = 50 1 month: −1.93 (−3.5 to −0.4) *p* = 014 6 months: −1.94 (−4.0 to 0.1) *p* = 06	G1 vs. G2 Pre: 0.46 (0.01 to 0.9) *p* = 0.05 1 month: −0.13 (-0.5 to 0.3) *p* = 0.53 6 months: −0.24 (-0.7 to 0.2) *p* = 0.24			G1 vs. G2 Pre: 0.17 (−0.3 to 0.7) *p* = 48 1 month: −0.09 (−0.6 to 0.4) *p* = 73 6 months: −0.49 (−1.0 to −0.01) *p* = 04		
	**Horng (2011)**	G1. Tendon gliding exercises + Paraffin + Night splint G2. Nerve gliding exercises + Paraffin + Night splint G3. Paraffin + Night splint	VAS G1 Pre vs. Post: *p* < 05 −19.7 ± 24.6	G2 Pre vs. Post: *p* < 05 −10.5 ± 18.0	G3 Pre vs. Post: *p* < 05 −17.2 ± 26.2	G1 vs. G2 vs. G3 *p* = 44	G1 Pre vs. Post: *p* < 05 −0.7 ± 0.8	G2 Pre vs. Post: *p* < 05 −0.3 ± 0.6	G3 Pre vs. Post: *p* < 05 −0.6 ± 0.6	G1 Pre vs. Post: *p* < 05 −0.4 ± 0.5	G2 Pre vs. Post: *p* > 05 0.1 ± 0.5	G3 Pre vs. Post: *p* > 05 −0.2 ± 0.7
							G1 vs. G2 vs. G3 *p* = 56			G1 vs. G2 vs. G3 *p* = 04		
	**Ordahan (2017)**	G1. Paraffin + Splint + Exercises G2. Splint + Exercises	VAS G1 Pre vs. Post: *p* = 0.009 7.31 ± 1.41 vs. 3.73 ± 1.19	G2 Pre vs. Post: *p* = 0.013 6.95 ± 1.60 vs. 3.80 ± 1.67		G1 vs. G2 *p* = 423	G1 Pre vs. Post: *p* = 011 3.07 ± 0.63 vs. 2.52 ± 0.59		G2 Pre vs. Post: *p* = 018 2.83 ± 0.68 vs. 2.52 ± 0.71	G1 Pre vs. Post: *p* = 027 2.83 ± 0.90 vs. 2.32 ± 0.89	G2 Pre vs. Post: *p* = 214 2.41 ± 0.83 vs. 2.42 ± 1.05	
							G1 vs. G2 *p* = 0.551			G1 vs. G2 *p* = 0.021		
	**Tezel (2019)**	G1. Acupuncture + Night splint G2. Night splint	VAS G1 Pre vs. Post: 7.4 ± 0.8 vs. 4.8 ± 0.8	G2 Pre vs. Post: 7.6 ± 0.7 vs. 5.8 ± 0.8		G1 vs. G2 *p* = 0.007	G1 Pre vs. Post: 29.8 ± 5.9 vs. 23.4 ± 7.5		G2 Pre vs. Post: 28.8 ± 5.4 vs. 22.1 ± 6.5	G1 Pre vs. Post: 26.8 ± 9.0 vs. 20.7 ± 6.9	G2 Pre vs. Post: 25.8 ± 8.7 vs. 19.4 ± 6.4	
							G1 vs. G2 *p* = 0.54			G1 vs. G2 *p* = 0.51		
**Combined**	**Atthakomol (2018)**	G1. Radial extracorporeal shock waves G2. Local corticosteroid injections	VAS G1 Pre: 2.4 ± 2.5 1 week: 1.3 ± 2.0 (*p* < 0.18) 4 weeks: 1.3 ± 1.9 (*p* < 0.15) 12 weeks: 0.65 ± 1.2 (*p* < 0.022) 24 weeks: 0.35 ± 0.81 (*p* < 0.0075)	G2 Pre: 2.6 ± 2.0 1 week: 1.6 ± 1.7 (*p* < 0.08) 4 weeks: 1.3 ± 1.5 (*p* < 0.08) 12 weeks: 1.9 ± 2.7 (*p* < 0.52) 24 weeks: 1.7 ± 2.1 (*p* < 0.19)		G1 vs. G2 Pre vs. 1w: −10 (−1.7 to 1.5) *p* = 90 1w vs. 4w: 0.049 (−1.6 to 1.7) *p* = 95 4w vs. 12w: −1.0 (−2.7 to 0.63) *p* = 23 12w vs. 24w: −1.2 (−2.9 to 0.49) *p* = 17	G1 Pre: 21 ± 6.4 1 week: 19 ± 7.4(*p* = 33) 4 weeks: 17 ± 4.3 *p* = 031) 12 weeks: 15 ± 4.5 (*p* = 0.0082) 24 weeks: 13 ± 2.9 (*p* = 0.0059)		G2 Pre: 22 ± 5.1 1 week: 17 ± 4.5 (*p* = 0.0047) 4 weeks: 17 ± 5.1 (*p* = 011) 12 weeks: 18 ± 5.5 (*p* = 13) 24 weeks: 19 ± 7.9 (*p* = 20)	G1 Pre: 14 ± 3.2 1 week: 13 ± 4.2 (*p* = 27) 4 weeks: 13 ± 3.5 (*p* = 12) 12 weeks: 11 ± 3.0 (*p* = 0.0065) 24 weeks: 11 ± 2.2 (*p* = 0.0073)	G2 Pre: 12 ± 4.1 1 week: 11 ± 3.2 (*p* = 31) 4 weeks: 11 ± 3.3 (*p* = 39) 12 weeks: 10 ± 3.4 (*p* = 19) 24 weeks: 13 ± 7.0 (*p* = 65)	
							G1 vs. G2 Pre vs. 1w: 2.6 (−2.0 to 7.2) *p* = 27 1w vs. 4w: 1.5 (−3.2 to 6.1) *p* = 53 4w vs. 12w: −1.9 (−2.7 to 2.8) *p* = 43 12w vs. 24w: −5.1 (−9.8 to −33) *p* = 036			G1 vs. G2 Pre vs. 1w: 0.43 (−3.1 to 3.9) *p* = 81 1w vs. 4w: −20 (−3.7 to 3.3) *p* = 91 4w vs. 12w: −1.8 (−5.3 to 1.7) *p* = 32 12w vs. 24w: −4.5 (−8.1 to −87) *p* = 015		
	**Incebiyik (2015)**	G1. Short wave diathermy + Gliding exercises G2. Placebo + Gliding exercises	VAS G1 Pre vs. Post: *p* < 0.001 5.50 ± 2.53 vs. 2.32 ± 1.80	G2 Pre vs. Post: *p* = 1.105 4.83 ± 2.76 vs. 4.20 ± 2.53		G1 vs. G2 *p* = 0.003	G1 Pre vs. Post: *p* < 0.001 30.78 ± 7.92 vs. 18.53 ± 9.09		G2 Pre vs. Post: *p* = 204 29.25 ± 11.41 vs. 27.62 ± 10.63	G1 Pre vs. Post: *p* < 0.001 29.8 ± 5.9 vs. 23.4 ± 7.5	G2 Pre vs. Post: *p* = 234 28.8 ± 5.4 vs. 22.1 ± 6.5	
							G1 vs. G2 **p* = 0.002*			G1 vs. G2 *p* < 0.001		
	**Talebi (2018)**	G1: Manual therapy–Nerve + interface mobilization + neural G2: TENS + Ultrasound	*VAS* G1 Pre vs. Post: *p = 0.000* 7.08 ± 1.56 vs. 3.75 ± 2.22	G2 Pre vs. Post: *p* = 0.000 6.58 ± 1.37 vs. 4.4 1 ± 1.31		G1 vs. G2 *p* = 141	G1 Pre vs. Post: *p* = 0.000 29.91 ± 9.65 vs. 19.25 ± 6.25		G2 Pre vs. Post: *p* = 0.000 29.91 ± 7.24 vs. 25.41 ± 6.25	G1 Pre vs. Post: *p* = 241 4.83 ± 2.76 vs. 4.20 ± 2.53	G2 Pre vs. Post: *p* = 0.008 4.83 ± 2.76 vs. 4.20 ± 2.53	
							G1 vs. G2 *p* = 0.006			G1 vs. G2 *p* = 043		
	**Wolny (2017)**	G1: Neurodynamic techniques + Function massage + Joint mobilization G2: Laser + ultrasound	*NPRS* G1 Pre vs. Post: *p* < 01 5.72 ± 1.49 vs. 1.47 ± 1.20	G2 Pre vs. Post: *p* < 01 5.25 ± 1.75 vs. 3.58 ± 1.93		G1 vs. G2 *p* < 01	G1 Pre vs. Post: 2.97 ± 0.63 vs. 1.78 ± 0.47		G2 Pre vs. Post: 2.94 ± 0.74 vs. 2.57 ± 0.77	G1 Pre vs. Post: 2.80 ± 0.94 vs. 1.90 ± 0.62	G2 Pre vs. Post: 2.77 ± 0.94 vs. 2.55 ± 0.95	
							G1 vs. G2 *p* < 0.001			G1 vs. G2 *p* < 0.001		
	**Wolny (2019)**	G1: Neurodynamic techniques G2: No treatment	*NPRS* G1 Pre vs. Post: 5.86 ± 1.46 vs. 1.38 ± 0.72	G2 Pre vs. Post: 5.71 ± 1.34 vs. 5.46 ± 1.05		G1 vs. G2 *p* < 0.001	G1 Pre vs. Post: 3.03 ± 0.65 vs. 1.08 ± 0.68		G2 Pre vs. Post: 2.92 ± 0.71 vs. 2.87 ± 0.68	G1 Pre vs. Post: 2.82 ± 0.71 vs. 1.96 ± 0.64	G2 Pre vs. Post: 2.99 ± 0.67 vs. 2.87 ± 0.71	
							G1 vs. G2 **p* < 0.001*			G1 vs. G2 *p* < 0.001		
	**Xu (2020)**	G1: Radial extracorporeal shock waves G2: Betamethasone 1mL	*VAS* G1 Pre: 2.5 ± 0.3 3 week: 1.4 ± 0.9(*p* = 0.04) 9 weeks:8 ± 1.1 (*p* = 0.02) 12 weeks: 0.6 ± 0.7 (*p* = 0.00)	G2 Pre: 2.6 ± 0.4 *3* week: 1.5 ± 1.1(*p* = 0.04) *9* weeks: 1.7 ± 0.7 (*p* = 0.21) 12 weeks: 1.9 ± 1.3 (*p* = 0.17)		G1 vs. G2 3 week: *p* = 56 9 weeks: *p* = 0.00 12 weeks *p* = 0.00	G1 *Pre*: 34.1 ± 4.3 3 week: 30.2 ± 3.7 (*p* = 0.04) 9 weeks: 25.4 ± 4.1 (*p* = 0.01) 12 weeks: 22.3 ± 2.7 (*p* = 0.00)			G2 Pre: 34.7 ± 5.6 3 week: 28.1 ± 6.7 (*p* = 0.03) 9 weeks: 28.9 ± 6.8 (*p* = 0.05) 12 weeks: 31.8 ± 3.4 (*p* = 53)		
							G1 vs. G2–3 week: *p* = 43; 9 weeks: *p* = 0.00; 12 weeks *p* = 0.00					
	**Yildiz (2011)**	G1: Ultrasound + Gel with ketoprofen + Splint G2: Ultrasound + Gel with drugs + Splint G3: Placebo ultrasound + Splint	VAS G1 Pre: 5.76 ± 2.45 2weeks: 2.72 ± 2.07 8 weeks: 3.48 ± 2.74	G2 Pre: 4.96 ± 2.50 2weeks: 2.41 ± 2.43 8 weeks: 2.77 ± 2.74	G3 Pre: 2.5 ± 0.3 2weeks: 3.03 ± 1.96 8 weeks: 0.98 ± 1.65	G1 < G3 *p* = 0.002 G2 < G3 *p* = 0.004	G1 Pre: 2.88 ± 0.55 2weeks: 1.94 ± 0.57 8 weeks: 2.08 ± 0.82	G2 Pre: 2.96 ± 0.62 2weeks: 2.04 ± 0.61 8 weeks: 1.97 ± 0.65	G3 Pre: 2.93 ± 1.04 2weeks: 1.78 ± 0.75 8 weeks: 1.63 ± 0.73	G1 Pre: 2.73 ± 0.73 2weeks: 2.08 ± 0.78 8 weeks: 2.19 ± 0.89	G2 Pre: 2.56 ± 0.64 2weeks: 1.93 ± 0.55 8 weeks: 1.98 ± 0.78	G3 Pre: 2.79 ± 1.05 2weeks: 2.16 ± 0.80 8 weeks: 1.79 ± 0.80
**Other**	**Eftekharsadat (2018)**	G1: Lavender ointment + Night splint G2: Placebo ointment + Night splint	VAS G1 Pre vs. Post: *p* = 049 6.96 ± 1.30 vs. 3.58 ± 1.59	G2 Pre vs. Post: 6.12 ± 1.54 vs. 4.79 ± 2.36		G1 vs. G2 *p* < 0.001	G1 Pre vs. Post: *p* < 0.001 2.20 ± 0.48 vs. 1.59 ± 0.29			G2 Pre vs. Post: *p* < 0.001 2.18 ± 0.49 vs. 1.78 ± 0.53		
							G1 vs. G2 *p* = 0.003					

BCTQ: Boston Carpal Tunnel Questionnaire; BCTQ-SSS: BCTQ Symptom Severity Scale; BCTQ-FSS: BCTQ Function Severity Scale; NPRS: Numeric Pain Rating Scale; VAS: visual analogue scale.

**Table 5:** PEDro Scale.

Study	1	2	3	4	5	6	7	8	9	10	11	Total
Atthakomol (2018)	X	X	X	X			X			X	X	7
Çatalbas (2018)	X	X		X	X		X	X		X		7
Chen (2015)	X	X	X	X			X			X	X	7
Chung (2016)	X	X	X	X	X		X	X	X	X	X	10
Dinarvand (2017)	X	X		X			X	X		X		6
Eftekharsadat (2018)	X	X		X	X	X	X	X		X		8
Fusakul (2014)	X	X	X	X	X		X	X		X		8
Geler-Külcü (2016)	X	X	X	X	X		X	X		X		8
Günay (2015)	X	X	X	X			X			X	X	7
Güner (2018)	X	X		X	X		X	X		X		7
Hadianfard (2015)	X	X		X			X	X	X	X	X	8
Hamzeh (2020)	X	X	X	X			X			X	X	7
Horng (2011)	X	X	X	X			X			X		6
Incebiyik (2015)	X	X	X	X	X		X			X	X	8
Karimzadeh (2019)	X	X		X	X			X		X		7
Kumnerddee (2010)	X	X		X				X		X	X	6
Ordahan (2017)	X	X		X			X	X		X		6
Raeissadat (2018)	X	X	X	X				X	X	X	X	8
Senna (2019)	X	X	X	X	X			X		X	X	8
Talebi (2018)	X	X		X			X	X	X	X	X	8
Tezel (2019)	X	X		X			X			X	X	6
Wang (2017)	X	X	X	X			X	X	X	X	X	9
Wolny (2019)	X	X	X	X			X			X		6
Wolny (2017)	X	X	X	X			X			X	X	7
Wu (2018)	X	X		X			X	X	X	X	X	8
Wu (2017)	X	X	X	X			X	X	X	X	X	9
Wu (2016)	X	X	X	X	X		X	X	X	X		9
Xu (2020)	X	X	X	X			X	X	X	X		8
Yildiz (2011)	X	X	X	X	X		X		X	X		8
Mean												7.5
